# Supplementary material for: Comprehensive analysis of RNA–chromatin, RNA–, and DNA–protein interactions
Source: NAR Genom Bioinform. 2025 Feb 24;7(1):lqaf010. doi: 10.1093/nargab/lqaf010 (PMC11850300; doi:10.1093/nargab/lqaf010)
Supplement: lqaf010_Supplemental_Files [file lqaf010_supplemental_files.zip › Khlebnikov_Suppl_Notes_R3.pdf]

Supplementary Note 1. One-to-all and all-to-all data comparison on Malat1, Hdac2 and Meg3 RNAs

It has been shown previously that the consistency of one-to-all interactions and all-to-all interactions data is small. It may seem, however, that the triad construction approach, when valid and applied to data from both types of experiment, should produce consistent sets of contacts.

A comparative analysis was conducted on the DNA-parts of triad contacts constructed from all-to-all data of Red-C RNA-DNA interactions and one-to-all interactions of lncRNA Malat1, Meg3 and mRNA Hdac2. No common contacts (such that their DNA parts overlap) were identified for the Malat1 data. While this may be interpreted as an unfavorable outcome for the triad construction method, it is not unexpected. The lncRNA MALAT1 is involved in the formation of nuclear speckles (NS). NS have a rather variable protein composition [1]. On the other hand, most NS-specific proteins are not represented in our protein list because no ChIP-seq and/or RNA-protein interaction data are available for them. Furthermore, the stable interaction of NS with chromatin is not obvious.

MALAT1 is a highly expressed RNA that may also be subject to rapid degradation. The distribution of this RNA throughout the nucleus is not solely determined by its function; diffusion also plays a role. The diffusivity [2], [3] and constant variability of the protein interactome of Malat1 RNA may result in incomplete data on its interactions with both proteins and DNA. As previously stated, this incompleteness may result in the inaccurate filtering of actual contacts when constructing triads, which may have occurred in the case of the all-to-all data. Furthermore, the lack of data on RNA- and DNA-protein interactions with speckle marker proteins, and thus the inability to construct triads for them, may also be a contributing factor.

Conversely, when we examined different triads constructed on one-to-all and all-to-all data for the Meg3 RNA, we managed to obtain a significant correlation of DNA loci the aforementioned RNA interacts with (**Figure SN1A-B**). This was observed for triads mediated by the EZH2 (correlation = 0.667, p-value = 1.59^{-5}) and SUZ12 (correlation = 0.263, p-value = 3.76^{-50}) proteins (**Figure SN1C-F**). This is of note, because the components of the PRC2 complex are known to interact with Meg3 RNA [4], [5].

Supplementary Note 2. Constructed triads may prove orthologous

The presence of conserved RNA-DNA interactions between organisms provides strong support for our triad filtering method. We tested for conserved interaction triads between organisms by comparing the interaction triads constructed from EZH2, hnRNPK, SUZ12, and WDR5 PR- and PD-interaction data for human (K562) and mouse (mESC) cell lines, using available data. We searched for RNA orthologues that form triads in both mouse and human as bidirectional hits with a relaxed significance threshold (**Suppl. Table S20**) using ortho2align [6]. We found 11 such RNA pairs. Next, we lifted over the DNA loci in contact with these RNAs to the opposite genome and searched for the closest DNA segment of the orthologous RNA contact. The distances between the DNA fragments and the DNA fragments in the original genome are substantial (**Table SN1**). However, we identified 10 pairs of orthologous RNA contacts (**Table SN2**) whose DNA fragments are within 2 Mb of one another. Considering the previously mentioned experiment specifics and the issue of inaccurate genomic interval lifting between genomes, this result confirms the reliability of our obtained data.

The limited number of orthologous triads and their composition can be explained. Firstly, we analysed significantly different cell types, as there were no better datasets available for similar cell types. Additionally, data obtained for RNA-chromatin contacts is rather noisy and incomplete. Therefore, consistent orthologous triads may not be found. Lastly, non-coding RNAs evolve rapidly, so corresponding orthologs may simply be missed. Therefore, the orthologous triads found are only for RNAs with a large number of contacts.

References

[1] Lukasz Galganski, Martyna O. Urbanek, and Wlodzimierz J. Krzyzosiak. "Nuclear speckles: molecular organization, biological function and role in disease". In: *Nucleic Acids Research* 45.18 (Sept. 2017), pp. 10350–10368. ISSN: 1362-4962. DOI: 10.1093/nar/gkx759. URL: http://dx.doi.org/10.1093/nar/gkx759.

[2] Nina Zablowsky et al. "High Throughput FISH Screening Identifies Small Molecules That Modulate Oncogenic lncRNA MALAT1 via GSK3B and hnRNPs". In: *Non-Coding RNA* 9.1 (Jan. 2023), p. 2. ISSN: 2311-553X. DOI: 10.3390/ncrna9010002. URL: http://dx.doi.org/10.3390/ncrna9010002.

[3] Zhanbing Ma et al. "LncRNA expression profile during autophagy and Malat1 function in macrophages". In: *PLOS ONE* 14.8 (Aug. 2019). Ed. by Rajeev Samant, e0221104. ISSN: 1932-6203. DOI: 10.1371/journal.pone.0221104. URL: http://dx.doi.org/10.1371/journal.pone.0221104.

[4] Minoru Terashima et al. "MEG3 Long Noncoding RNA Contributes to the Epigenetic Regulation of Epithelial-Mesenchymal Transition in Lung Cancer Cell Lines". In: *Journal of Biological Chemistry* 292.1 (Jan. 2017), pp. 82–99. ISSN: 0021-9258. DOI: 10.1074/jbc.m116.750950. URL: http://dx.doi.org/10.1074/jbc.M116.750950.

[5] Tanmoy Mondal et al. "MEG3 long noncoding RNA regulates the TGF-β pathway genes through formation of RNA–DNA triplex structures". In: *Nature Communications* 6.1 (July 2015). ISSN: 2041-1723. DOI: 10.1038/ncomms8743. URL: http://dx.doi.org/10.1038/ncomms8743.

[6] Dmitry Evgenevich Mylarshchikov and Andrey Alexandrovich Mironov. "ortho2align: a sensitive approach for searching for orthologues of novel lncRNAs". en. In: *BMC Bioinformatics* 23.1 (Sept. 2022), p. 384.

[7] James T Robinson et al. "Integrative genomics viewer". In: *Nature Biotechnology* 29.1 (Jan. 2011), pp. 24–26. ISSN: 1546-1696. DOI: 10.1038/nbt.1754. URL: http://dx.doi.org/10.1038/nbt.1754.

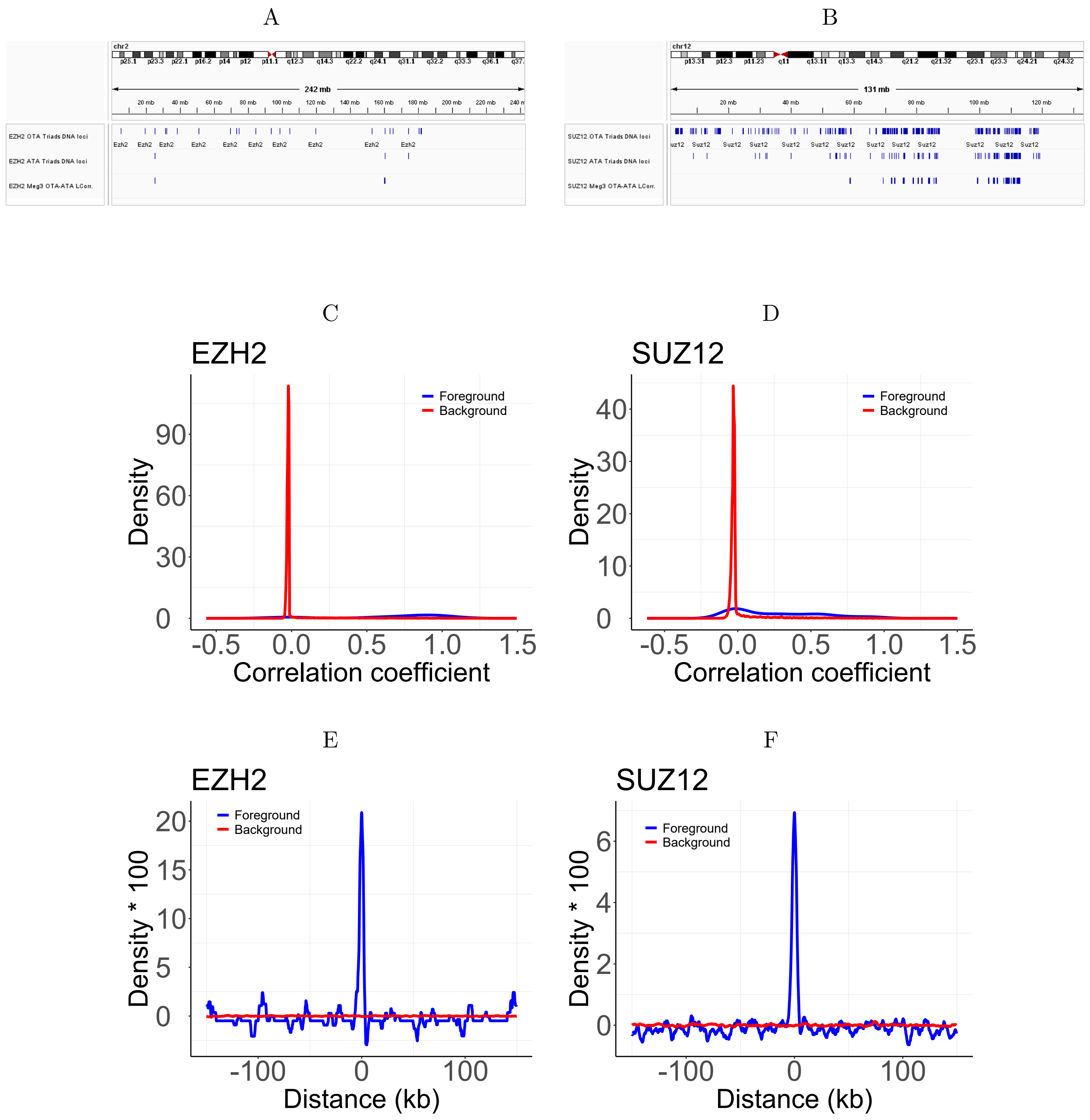

Figure SN1: One- and all-to-all data comparison for Meg3 lncRNA. An example of consistency between one-to-all and all-to-all approaches for EZH2 **(A)** and SUZ12 **(B)** triads in IGV [7]; Distribution of correlations for EZH2 **(C)** and SUZ12 **(D)** triads; Cross-correlation function decline for EZH2 **(E)** and SUZ12 **(F)** triads.

Table SN1: DNA contacts distances in original genomes for RNA orthologs. *"RNA from source genome"* stands for RNA, for which coordinates of DNA locus that it interacts with were unchanged. *"RNA for liftOver"* stands for RNA, for which coordinates of DNA locus were lifted over to the opposing genome.

DNA Chr	RNA from source genome	RNA for liftOver	Distance, nt	Triads formed by
chr16	Gm24299	SNORD5	71101	SUZ12
chr22	SNORD5	Gm24299	79851	SUZ12
chr16	Gm24265	RNU4-2	114378	EZH2
chr11	Rpph1	RPPH1	334071	HNRNPK
chr16	Gm24265	RNU4-2	409387	SUZ12
chr11	Gm24265	RNU4-2	627098	SUZ12
chr2	Rpph1	RPPH1	1529017	EZH2
chr10	Gm24299	SNORD5	1575789	SUZ12
chr12	SNORD5	Gm24299	1722366	SUZ12
chr19	RNU4-2	Gm24265	1920768	SUZ12
chr11	Rpph1	RPPH1	2543554	EZH2
chr7	RNU4-2	Gm24265	2644315	SUZ12
chr6	Gm24265	RNU4-2	2704283	SUZ12
chr12	Gm26247	C19orf48	4866317	EZH2
chr17	Gm50452	U79	5350920	EZH2
chr15	Rpph1	RPPH1	5502957	EZH2
chr14	C19orf48	Gm26247	6136940	EZH2
chr17	RNU4-2	Gm24265	6459825	SUZ12
chr6	U79	Gm50452	6513447	EZH2
chr12	RPPH1	Rpph1	6675254	EZH2
chr2	Gas5	U79	7172769	EZH2
chr6	RPPH1	Rpph1	7203675	EZH2
chr3	Rpph1	RPPH1	7558544	EZH2
chr15	Gm24265	RNU4-2	7737350	SUZ12
chr7	RNU4-2	Gm24265	8161423	SUZ12
chr2	U79	Gas5	8529496	EZH2

See next page

Supplementary Table SN1 – continued from previous page

DNA Chr	RNA from source genome	RNA for liftOver	Distance, nt	Triads formed by
chr22	RPPH1	Rpph1	9440164	EZH2
chr2	U79	Gas5	9528528	EZH2
chr10	Rpph1	RPPH1	9815790	EZH2
chr17	Rpph1	RPPH1	10880342	EZH2
chr5	RPPH1	Rpph1	11601727	EZH2
chr2	Rpph1	RPPH1	11693085	EZH2
chr10	RPPH1	Rpph1	12013038	EZH2
chr2	RPPH1	Rpph1	12542982	EZH2
chr16	Gm24265	RNU4-2	12618598	SUZ12
chr5	Rpph1	RPPH1	13398672	EZH2
chr11	Gm23301	SNORD58B	13574192	HNRNPK
chr11	Gm24265	RNU4-2	17752338	SUZ12
chr16	Gm50452	U79	18906235	EZH2
chr12	RPPH1	Rpph1	19771353	EZH2
chr22	RNU4-2	Gm24265	20303830	SUZ12
chr7	SNORD58B	Gm23301	20517250	EZH2
chr8	Gm24265	RNU4-2	20957564	SUZ12
chr7	RNU4-2	Gm24265	21869558	SUZ12
chr11	Gm23301	SNORD58B	22013524	EZH2
chr3	RPPH1	Rpph1	23617849	EZH2
chr10	U79	Gm50452	24060208	EZH2
chr1	Gm23301	SNORD58B	26949946	EZH2
chr8	Rpph1	RPPH1	28768814	EZH2
chr17	SNORD58B	Gm23301	30342819	EZH2
chr2	Gas5	U79	33594729	EZH2
chr14	Gm24265	RNU4-2	34242552	SUZ12
chr18	Gm23301	SNORD58B	34937500	EZH2

See next page

Supplementary Table SN1 – continued from previous page

DNA Chr	RNA from source genome	RNA for liftOver	Distance, nt	Triads formed by
chr11	RPPH1	Rpph1	35518520	EZH2
chr17	Gm24265	RNU4-2	39573176	SUZ12
chr11	Rpph1	RPPH1	40362990	EZH2
chr7	Rpph1	RPPH1	41092890	EZH2
chr9	Gm50452	U79	44981079	EZH2
chr6	Gm24299	SNORD5	45606058	SUZ12
chr4	Gm50452	U79	46787576	EZH2
chr7	SNORD5	Gm24299	51881334	SUZ12
chr2	Rpph1	RPPH1	52531323	EZH2
chr3	Rpph1	RPPH1	55440322	EZH2
chr16	Gm24265	RNU4-2	58216738	SUZ12
chr2	Rpph1	RPPH1	60112309	EZH2
chr20	U79	Gas5	62122460	EZH2
chr16	RNU4-2	Gm24265	66900238	SUZ12
chr17	Rpph1	RPPH1	70320379	EZH2
chr6	RNU4-2	Gm24265	74855836	SUZ12
chr5	Gm50452	U79	88642071	EZH2
chr1	Gas5	U79	103951727	EZH2
chr5	Gm50452	U79	114030242	EZH2
chr1	Rpph1	RPPH1	114229243	EZH2
chr7	U79	Gm50452	131126867	EZH2

Table SN2: Top 10 potentially orthologous protein-mediated RNA-DNA contacts with the closest distance. Distance is defined as the distance between the locus in the mouse genome and the locus lifted from the human genome to the mouse genome or vice versa.

DNA Chr	Mouse RNA	Human RNA	Distance	Protein	Closest gene	
					Mouse gene	Human gene
chr16	Gm24299	SNORD5	71 101	SUZ12	Lrrc74b, P2rx6	XR_933144.3, XYLT1
chr22	Gm24299	SNORD5	79 851	SUZ12	Pi4ka, Serpind1	AIFM3
chr16	Gm24265	RNU4-2	114 378	EZH2	Rimbp3, Hic2	XYLT1
chr11	Rpph1	RPPH1	334 071	hnRNPK	Tex14, Rnu3b4	TMEM135
chr16	Gm24265	RNU4-2	409 387	SUZ12	Rimbp3, Hic2	No close genes
chr11	Gm24265	RNU4-2	627 098	SUZ12	Rnf213	No close genes
chr2	Rpph1	RPPH1	1 529 017	EZH2	Gm40034	No close genes
chr10	Gm24299	SNORD5	1 575 789	SUZ12	Cyp27b1, Mettl1, Marchf9	No close genes
chr12	Gm24299	SNORD5	1 722 366	SUZ12	Ttc6	RPS26
chr19	Gm24265	RNU4-2	1 920 768	SUZ12	Ms4a4a	CACNA1A
