# Supplementary material for: Comprehensive analysis of RNA–chromatin, RNA–, and DNA–protein interactions
Source: NAR Genom Bioinform. 2025 Feb 24;7(1):lqaf010. doi: 10.1093/nargab/lqaf010 (PMC11850300; doi:10.1093/nargab/lqaf010)
Supplement: lqaf010_Supplemental_Files [file lqaf010_supplemental_files.zip › Khlebnikov_Suppl_Materials_R2.pdf]

Supplementary Materials

Table S1:	RNA-DNA contacts RNA biotypes contents.	3
Table S2:	Proteins split into three groups according to their putative function inferred from UniProt.	3
Table S3:	Database identifiers for data used in the study. IDs correspond to databases the following way: GSE.../GSM... – NCBI GEO, ENCS... – ENCODE, PEAKS... – GTRD, ERP... – ArrayExpress.	4
Table S4:	RNA-protein peak statistics, total number and mRNA peaks content.	7
Table S5:	BaRDIC running parameters for peak calling in all- and one-to-all RNA-DNA data.	9
Table S6:	Triad number magnitudes when applying peak extension procedure.	9
Table S7:	Grouped ChromHMM annotation states.	10
Table S8:	Grouped SPIN annotation states.	10
Table S9:	StereoGene parameters changed from defaults when correlating DNA tracks	11
Table S10:	Distribution of the of cis- and trans-contacts abundances in constructed triads for each protein. Unless stated otherwise, triads are based on eCLIP RNA-protein interactions data, Red-C RNA-DNA interactions data for K562 cells. Ratios of mRNA-mediated triads are also shown.	11
Table S11:	cis-/trans- contacts ratio statistics for selected RNAs for K562 Red-C experiment.	14
Table S12:	Triads and ChIP-seq composition by ChromHMM annotation of DNA loci for all proteins split by function. *cnt* – number of observed ChIP-seq loci in a state; *tri* – number of triads; Z – z-score of $\frac{tri}{cnt}$ distribution over all ChromHMM states	15
Table S13:	Triads and RD-contacts DNA parts composition by ChromHMM annotation of DNA loci for all proteins split by function. *cnt* – number of observed DNA-parts in a state; *tri* – number of triads; Z – z-score of $\frac{tri}{cnt}$ distribution over all ChromHMM states	16
Table S14:	Triads and ChIP-seq composition by SPIN annotation of DNA loci for all proteins split by function. *cnt* – number of observed ChIP-seq loci in a state; *tri* – number of triads; Z – z-score of $\frac{tri}{cnt}$ distribution over all SPIN states	16
Table S15:	Triads and and RD-contacts DNA parts composition by SPIN annotation of DNA loci for all proteins split by function. *cnt* – number of of observed DNA-parts in a state; *tri* – number of triads; Z – z-score of $\frac{tri}{cnt}$ distribution over all SPIN states	17
Table S16:	Triads and and RD-contacts composition by RNA biotypes for all proteins split by function. *cnt* – number of of observed RNA of a given biotype; *tri* – number of triads; Z – z-score of $\frac{tri}{cnt}$ distribution over all RNA biotypes	18
Table S17:	-log10(p-values) for the real number of triads on the background distribution of the number of triads obtained on simulated data. The background distribution for triads number was fit using a gamma distribution, and the p-value defined as the probability of obtaining the real number of triads for protein or a value greater from the fit distribution.	18
Table S18:	Results for the association tests: χ^2- goodness-of-fit of ChromHMM annotation of DNA-parts and Fisher's exact test for association of PR- and PD-contacts data of protein with RD-contacts.	21
Table S19:	Results for the association test: χ^2- goodness-of-fit of SPIN annotation of DNA-parts of triads and proteins ChIP-seq loci.	22
Table S20:	RNA orthologs found by ortho2align (selected as bidirectional best hit)	24
Figure S1:	Threshold selection for triads construction filtering using size of intersection between peaks and correspondent part of an RNA-DNA contact.	25
Figure S2:	Protein clusterization based on their "related" proteins	25
Figure S3:	Circos diagrams for constructed protein mediated RNA-DNA interactions, or triads. Legend for diagrams is shown in the main text.	30
Figure S4:	Ridgeline plot for number of simulated triads for chromatin remodelling proteins.	31
Figure S5:	Ridgeline plot for number of simulated triads for RNA processing and export proteins.	32
Figure S6:	Ridgeline plot for number of simulated triads for transcription regulating proteins.	33
Figure S7:	Barplots displaying the number of simulated triads for each functional group of proteins.	33
Figure S8:	Strand cross-correlation for WDR5 ChIP-seq.	34

Figure S9: The RADICL-seq data for various proteins and experimental phases, namely 2FA, NPM, and 2FA-no-NPM (representing triads present in 2FA but not in NPM). **(A-C)** The ratio of shared contacts in triads for the RADICL-seq 2FA experiment **(A)**, RADICL-seq NPM experiment **(B)**, and RADICL-seq 2FA without RADICL-seq NPM **(C)** is shown. The heatmaps are asymmetrical because of the normalization process by RDt-contacts set magnitude. This means that the magnitudes of horizontal triads datasets is divided by the magnitudes of the corresponding datasets on the vertical axis. Values greater than 1 are coloured the same as those equal to 1. If a value here is greater than 1, this means that the horizontal triads dataset is a subset of a vertical one. **(D-F)** Correlation coefficients of DNA-parts tracks of the contacts for the RADICL-seq 2FA experiment **(D)**, RADICL NPM experiment **(E)**, and RADICL-seq 2FA without RADICL-seq NPM **(F)** are shown. **(G, H)** RDt-scaling plots for cis-contacts of the RADICL-seq experiment **(G)** and for RADICL-seq-based triads **(H)**. **(I)** Ratios of ChromHMM states distribution of DNA-parts for RADICL-seq 2FA, RADICL-seq NPM and RADICL-seq 2FA without RADICL-seq NPM experiments. 35
Figure S10: Triads number desaturation when filtering the 2FA contacts shared with NPM phase. 36
Figure S11: Number of triads simulated for RADICL-seq 2FA and RADICL-seq NPM datasets differs significantly with the latter number being larger. 37
Figure S12: Comparison of SUZ12-mediated triads based on eCLIP- and fRIP-seq PR-data. **(A, B)** Euler's diagrams for common RNAs **(A)** and shared RD-contacts **(B)** for these datasets (a contact from one dataset is considered shared if its DNA-part intersects with any DNA-part of at least one contact in the opposing dataset and RNA-parts of these contacts belong to the same transcript). **(C)** RNA biotype composition comparison of the transcripts from which the RNA parts of triads originated. **(D)** Ratios of ChromHMM states distribution for DNA-parts of SUZ12-mediated triads based on eCLIP and fRIP-seq data. 38
Figure S13: Biotype content of RNAs forming triads in all fRIP-based triads **(A)**, all eCLIP-based triads **(B)**, triads shared by two experiments **(C)** and triads present only in one of datasets **(D)**. . . 39
Figure S14: SUZ12 DNA-parts tracks correlation. **(A)** Distribution of correlations across all bins; **(B)** Cross-correlation function decline. blue — observed correlation; red — background correlations 40
Figure S15: Common RNA between contacts of RADICL-seq and GRID-seq in triads formed by EZH2 **(A)**, hnRNPK **(B)**, SUZ12 **(C)** and WDR5 **(D)** proteins. 40
Figure S16: Consistency analysis for EZH2-mediated triads constructed on RADICL-seq and GRID-seq data. While we can't ensure the completeness of both datasets due to experimental protocols specifics, triads shared between two experiments might be interesting to study. It is important to note, however, that the consistent triads and real EZH2-mediated triads sets might also have no objects in common. 41
Figure S17: Stacked barplots and paired barplots representing DNA parts distribution along the ChromHMM annotation states for triads of proteins in both cell lines. 45
Figure S18: Stacked barplots and paired barplots representing DNA parts distribution along the SPIN annotation states for triads of proteins in both cell lines. 49
Figure S19: DNA parts of triads distribution by ChromHMM states obtained using real and simulated DNA- and RNA-protein interaction data for RNA processing and exporting proteins. 50
Figure S20: DNA parts of triads distribution by ChromHMM states obtained using real and simulated DNA- and RNA-protein interaction data for RNA processing and exporting proteins. Proteins are grouped so that there are no intersections between groups. 50
Figure S21: DNA parts of triads distribution by SPIN states obtained using real and simulated DNA- and RNA-protein interaction data. 51
Figure S22: DNA parts of triads distribution by SPIN states obtained using real and simulated DNA- and RNA-protein interaction data. Proteins are grouped so that there are no intersections between groups. 51
Figure S23: RNA participation in triads, i.e. the possible range of proteins number an RNA forms triads with. 52

Supplementary Table S1. RNA-DNA contacts RNA biotypes contents.

RD protocol		lincRNA	miRNA	protein coding	snoRNA	snRNA	rRNA	vlinc RNA	X-RNA	pseudo-gene	other
GRID-seq mESC	total	1907127	57107	434656	3009	514285	4325953	0	7188737	61476817	3770432
	cis	402475	4629	179511	1750	29933	174576	0	909216	30336481	353721
	trans	1504652	52478	255145	1259	484352	4151377	0	6279521	31140336	3416711
RADICL-seq 2FA mESC	total	1474496	244487	34603158	1256074	1685073	8344158	0	7009281	58070	862696
	cis	214810	10727	14059007	72624	65660	331613	0	511121	14323	157724
	trans	1259686	233760	20544151	1183450	1619413	8012545	0	6498160	43747	704972
RADICL-seq NPM mESC	total	735019	56880	19104867	1382997	295703	19743288	0	4725658	69803	635440
	cis	22574	2439	979501	75434	16203	740136	0	205064	3615	32811
	trans	712445	54441	18125366	1307563	279500	19003152	0	4520594	66188	602629
Red-C K562	total	6056433	5820	41421379	477631	513445	586235	2308138	2027429	785617	485239
	cis	863404	169	7462332	21846	26889	13686	464762	413353	113467	29371
	trans	5193029	5651	33959047	455785	486556	572549	1843376	1614076	672150	455868
Red-ChIP CTCF K562	total	3225931	16460	23567457	371165	527081	142219	1298088	1489978	349501	271866
	cis	607831	2629	5923288	20076	23423	6172	329077	401896	68808	22146
	trans	2618100	13831	17644169	351089	503658	136047	969011	1088082	280693	249720
Red-ChIP Input K562	total	3478995	20589	25950921	488346	610157	257215	1396607	1606358	395175	329189
	cis	628760	2937	6185907	25042	30880	8454	339759	404909	73634	26321
	trans	2850235	17652	19765014	463304	579277	248761	1056848	1201449	321541	302868

Supplementary Table S2. Proteins split into three groups according to their putative function inferred from UniProt.

Protein group	Grouped proteins with intersections	Grouped proteins without intersections
Chromatin modification and remodelling	CBP, CBX3, CHD1, CHD4, CHD7, CTCF, DNMT1, EZH2, HDAC1, HLTF, LSD1, MORC2, PCAF, PHF8, RBBP5, SAFB, SAFB2, SUZ12, WDR5	CBP, CBX3, CHD1, CHD4, CHD7, CTCF, DNMT1, EZH2, HDAC1, HLTF, LSD1, MORC2, PCAF, PHF8, RBBP5, SAFB, SAFB2, SUZ12, WDR5

See next page

Supplementary Table S2 – continued from previous page

Protein group	Grouped proteins with intersections	Grouped proteins without intersections
RNA processing and export	FUS, hnRNPC, hnRNPH, hnRNPL, hnRNPUL1, ILF3, KHSRP, NONO, NUP98, PTBP1, RBFOX2, RBM15, RBM22, SRSF1, SRSF7, SRSF9, TARDBP, U2AF1, U2AF2, ZC3H11A	FUS, hnRNPC, hnRNPH, hnRNPL, hnRNPUL1, ILF3, KHSRP, NONO, NUP98, PTBP1, RBFOX2, RBM15, RBM22, SRSF1, SRSF7, SRSF9, TARDBP, U2AF1, U2AF2, ZC3H11A
Transcription regulation	EWSR1, FUS, GTF2F1, hnRNPK, hnRNPUL1, LARP7, NONO, PCBP1, TAF15, U2AF2, YBX3, ZC3H8	EWSR1, GTF2F1, hnRNPK, LARP7, PCBP1, TAF15, YBX3, ZC3H8

Supplementary Table S3. Database identifiers for data used in the study. IDs correspond to databases the following way: GSE. . . /GSM. . . – NCBI GEO, ENCS. . . – ENCODE, PEAKS. . . – GTRD, ERP. . . – ArrayExpress.

Protein	RNA-protein data	ChIP-seq data
CBP	K562 fRIP: GSE67963	K562: ENCSR000ATT
CBX3	K562 fRIP: GSE67963	K562: ENCSR000ATV
CHD1	K562 fRIP: GSE67963	K562: ENCSR000AQD
CHD4	K562 fRIP: GSE67963	K562: ENCSR000ATL
CHD7	K562 fRIP: GSE67963	K562: ENCSR000AVD
CTCF	K562 fRIP: GSE67963	K562: ENCSR000AKO
DNMT1	K562 fRIP: GSE67963	K562: ENCSR987PBI
EWSR1	K562 eCLIP: ENCSR887LPK	K562: ENCSR142YYA
EZH2	K562 fRIP: GSE67963; mESC eCLIP: GSE141700	K562: ENCSR000AQE; mESC: PEAKS042326
FUS	K562 eCLIP: ENCSR069EVH	K562: ENCSR051DXE
GTF2F1	K562 eCLIP: ENCSR736AAG	K562: ENCSR000EHC
HDAC1	K562 fRIP: GSE67963	K562: ENCSR000AQF

See next page

Protein	RNA-protein data	ChIP-seq data
HLTF	K562 eCLIP: ENCSR589YHM	K562: ENCSR090JNM
hnRNPC	K562 eCLIP: ENCSR249ROI	K562: PEAKS057732
hnRNPH	K562 fRIP: GSE67963	K562: GSE120104
hnRNPK	K562 eCLIP: ENCSR268ETU; mESC eCLIP: GSE87233	K562: ENCSR014RCS; mESC: GSE129471
hnRNPL	K562 eCLIP: ENCSR795CAI	K562: ENCSR594BNR
hnRNPUL1	K562 eCLIP: ENCSR571VHI	K562: ENCSR296MXW
ILF3	K562 eCLIP: ENCSR438KWZ	K562: ENCSR632TJQ
KHSRP	K562 eCLIP: ENCSR438GZQ	K562: ENCSR686EYO
LARP7	K562 eCLIP: ENCSR456KXI	K562: ENCSR288MOZ
LSD1	K562 fRIP: GSE67963	K562: PEAKS048159, PEAKS048160
MORC2	K562 eCLIP: ENCSR168VUE	K562: GSE95374
NONO	K562 eCLIP: ENCSR861PAR	K562: ENCSR010KFT
NUP98	K562 fRIP: GSE67963	K562: PEAKS048332
PCAF	K562 fRIP: GSE67963	K562: ENCSR000ATZ
PCBP1	K562 eCLIP: ENCSR922WJV	K562: ENCSR052PTN
PHF8	K562 fRIP: GSE67963	K562: ENCSR000AQH
PTBP1	K562 eCLIP: ENCSR981WKN	K562: ENCSR948KMB
RBBP5	K562 fRIP: GSE67963	K562: ENCSR000AQI

See next page

Supplementary Table S3 – continued from previous page

Protein	RNA-protein data	ChIP-seq data
RBFOX2	K562 eCLIP: ENCSR756CKJ	K562: ENCSR822LBD
RBM15	K562 eCLIP: ENCSR196INN	K562: ENCSR850WUE
RBM22	K562 eCLIP: ENCSR295OKT	K562: ENCSR848AOP
SAFB	K562 eCLIP: ENCSR484LAB	K562: ENCSR072VUO
SAFB2	K562 eCLIP: ENCSR943MHU	K562: ENCSR724GSW
SRSF1	K562 eCLIP: ENCSR432XUP	K562: ENCSR832DMO
SRSF7	K562 eCLIP: ENCSR468FSW	K562: ENCSR222MYK
SRSF9	K562 eCLIP: ENCSR381VNR	K562: ENCSR814AJH
SUZ12	K562 fRIP: GSE67963; mESC eCLIP: GSE141700; mESC fRIP: ERP113531	K562: ENCSR000AUC; mESC: PEAKS057610
TAF15	K562 eCLIP: ENCSR568DZW	K562: ENCSR047LSJ
TARDBP	K562 eCLIP: ENCSR720BJU	K562: ENCSR033VAZ
U2AF1	K562 eCLIP: ENCSR862QCH	K562: ENCSR690GUG
U2AF2	K562 eCLIP: ENCSR893RAV	K562: ENCSR479QAJ
WDR5	K562 fRIP: GSE67963; mESC fRIP: GSE53035;	K562: PEAKS050903, PEAKS050904; mESC: GSM566279
YBX3	K562 eCLIP: ENCSR529FKI	K562: ENCSR567JEU
ZC3H11A	K562 eCLIP: ENCSR712IAG	K562: ENCSR000EFR
ZC3H8	K562 eCLIP: ENCSR181NRW	K562: ENCSR494PWZ

Supplementary Table S4. RNA-protein peak statistics, total number and mRNA peaks content.

Protein, experiment and cell line	Total number of peaks	Number of mRNA peaks	Number of ChIP-seq peaks
CBP fRIP K562	16637	23336	787
CBX3 fRIP K562	17153	24417	752
CHD1 fRIP K562	20975	29579	11282
CHD4 fRIP K562	17574	24401	5621
CHD7 fRIP K562	17766	24677	1175
CTCF fRIP K562	23815	33150	49823
DNMT1 fRIP K562	15193	20959	3560
EWSR1 eCLIP K562	13100	22716	1257
EZH2 eCLIP mESC	21578	25873	1905
EZH2 fRIP K562	19004	26030	714
FUS eCLIP K562	4689	8426	2900
GTF2F1 eCLIP K562	12195	21560	4857
HDAC1 fRIP K562	9300	15250	17228
HLTF eCLIP K562	4300	7514	10568
HNRNPC eCLIP K562	2275	3297	1016
HNRNPH fRIP K562	10020	18012	209
HNRNPK eCLIP K562	21439	36106	12345
HNRNPK eCLIP mESC	21423	26257	630
HNRNPL eCLIP K562	5024	9959	8042
HNRNPUL1 eCLIP K562	13522	23277	1019
ILF3 eCLIP K562	5413	9140	3435
KHSRP eCLIP K562	14841	21291	4087
LARP7 eCLIP K562	20838	33168	7434
LSD1 fRIP K562	18838	24903	30551

See next page

Supplementary Table S4 – continued from previous page

Protein, experiment and cell line	Total number of peaks	Number of mRNA peaks	Number of ChIP-seq peaks
MORC2 eCLIP K562	29685	42026	4685
NONO eCLIP K562	11369	20254	10610
NUP98 fRIP K562	21277	28657	5405
PCAF fRIP K562	19459	25611	757
PCBP1 eCLIP K562	20923	32623	10971
PHF8 fRIP K562	15689	23958	29345
PTBP1 eCLIP K562	20659	35405	4887
RBBP5 fRIP K562	14214	22174	24762
RBFOX2 eCLIP K562	5143	8894	57046
RBM15 eCLIP K562	16680	25185	284
RBM22 eCLIP K562	30231	45151	12860
SAFB2 eCLIP K562	4865	12441	630
SAFB eCLIP K562	4250	11282	6170
SRSF1 eCLIP K562	12831	18690	256
SRSF7 eCLIP K562	18124	26327	210
SRSF9 eCLIP K562	29928	49158	2410
SUZ12 eCLIP mESC	14281	17443	21081
SUZ12 fRIP K562	13877	17999	2410
SUZ12 fRIP mESC	12136	15508	21081
TAF15 eCLIP K562	10893	20255	797
TARDBP eCLIP K562	17821	27009	12678
U2AF1 eCLIP K562	22924	33385	4434
U2AF2 eCLIP K562	19772	28047	131
WDR5 fRIP K562	19613	26623	13511
WDR5 fRIP mESC	1425	1700	13282
YBX3 eCLIP K562	6136	8760	1783

See next page

Supplementary Table S4 – continued from previous page

Protein, experiment and cell line	Total number of peaks	Number of mRNA peaks	Number of ChIP-seq peaks
ZC3H11A eCLIP K562	8143	13098	2484
ZC3H8 eCLIP K562	25360	39250	11043

Supplementary Table S5. BaRDIC running parameters for peak calling in all- and one-to-all RNA-DNA data.

Option	Value
`-min_contacts`	1000
`-trans_min`	1000
`-trans_max`	1000000
`-trans_step`	100
`-cis_min`	1.1
`-cis_max`	2
`-cis_step`	0.01
`-cis_start`	100
`-tolerance`	0.01
`-window`	1
`-ifactor`	0.01
`-degree`	3
`-max_threshold`	0.05
`-fill_value`	1
`-qval_threshold`	0.05
`-bgtype`	custom

Supplementary Table S6. Triad number magnitudes when applying peak extension procedure.

DNA extend both	RNA extend dowstream	TRIADS w mRNAs	TRIADS w/o mRNAs
0	0	257 749	210 681
0	50	263 862	214 112

See next page

Supplementary Table S6 – continued from previous page

DNA extend both	RNA extend dowstream	TRIADS w mRNAs	TRIADS w/o mRNAs
0	100	272 556	220 127
1000	0	1 271 470	1 038 752
1000	50	1 301 594	1 055 370
1000	100	1 343 721	1 084 776
2000	0	2 163 250	1 770 970
2000	50	2 214 383	1 799 471
2000	100	2 284 709	1 848 423

Supplementary Table S7. Grouped ChromHMM annotation states.

Grouped State	Original ChromHMM State
Promoter	Active Promoter, Weak Promoter, Inactive/poised Promoter
Enhancer	Strong enhancer, Weak/poised enhancer
Transcribed	Transcriptional transition, Transcriptional elongation, Weak transcription
Insulator	Insulator
PRC_Repressed	Polycomb-repressed
HetChrom_CNV	Heterochromatin; low signal, Repetitive/Copy Number Variation

Supplementary Table S8. Grouped SPIN annotation states.

Grouped State	Original SPIN State
A_compartment	Interior_Act1, Interior_Act2, Interior_Act3
B_compartment	Interior_Repr1, Interior_Repr2
Lamina	Lamina
Lamina_like	Lamina_like
Near_Lm	Near_Lm1, Near_Lm2
Speckle	Speckle

Supplementary Table S9. StereoGene parameters changed from defaults when correlating DNA tracks

Option	Value
`Rscript`	1
`wSize`	300000
`wStep`	300000
`maxNA`	99.9
`maxZero`	99.9
`kernelSigma`	2000
`bin`	200
`outLC`	1

Supplementary Table S10. Distribution of the of cis- and trans-contacts abundances in constructed triads for each protein. Unless stated otherwise, triads are based on eCLIP RNA-protein interactions data, Red-C RNA-DNA interactions data for K562 cells. Ratios of mRNA-mediated triads are also shown.

Protein	Cis-contacts number	Trans-contacts number	mRNA contacts ratio
CBP fRIP	26	497	0.4608
CBX3 fRIP	136	745	0.4721
CHD1 fRIP	856	10396	0.4878
CHD4 fRIP	200	2953	0.5839
CHD7 fRIP	99	782	0.6139
CTCF fRIP	2368	29571	0.4818
DNMT1 fRIP	52	715	0.6698
EWSR1	157	1045	0.3135
EZH2 GRID-seq mESC	664	3480	0.0443
EZH2 RADICL-seq 2FA mESC	2965	21016	0.1435
EZH2 RADICL-seq NPM mESC	2976	35917	0.0799
EZH2 fRIP	44	710	0.6370
FUS	124	95	0.6451

See next page

Supplementary Table S10 – continued from previous page

Protein	Cis-contacts number	Trans-contacts number	mRNA contacts ratio
GTF2F1	238	2173	0.3867
HDAC1 fRIP	522	5490	0.5099
HLTF	46	1220	0.3788
HNRNPC	10	17	0.4490
HNRNPH fRIP	22	36	0.5538
HNRNPK GRID-seq mESC	16	153	0.1592
HNRNPK RADICL-seq 2FA mESC	162	1423	0.1587
HNRNPK RADICL-seq NPM mESC	76	2879	0.0817
HNRNPK	631	7096	0.3692
HNRNPL	57	323	0.4501
HNRNPUL1	243	1451	0.2754
ILF3	23	562	0.1667
KHSRP	28	145	0.7882
LARP7	725	7210	0.3677
LSD1 fRIP	1359	20011	0.4914
MORC2	98	1802	0.1453
NONO	239	1974	0.5931
NUP98 fRIP	290	4153	0.4960
PCAF fRIP	29	321	0.6881
PCBP1	864	9524	0.4099
PHF8 fRIP	1931	20136	0.4601
PTBP1	497	4780	0.3508
RBBP5 fRIP	1362	19515	0.4110
RBFOX2	257	1169	0.5750

See next page

Supplementary Table S10 – continued from previous page

Protein	Cis-contacts number	Trans-contacts number	mRNA contacts ratio
RBM15	15	229	0.4106
RBM22	772	10296	0.3857
SAFB2	14	36	0.1935
SAFB	122	480	0.3348
SRSF1	25	185	0.6111
SRSF7	4	81	0.5874
SRSF9	5	21	0.0370
SUZ12 GRID-seq mESC	3899	56261	0.0987
SUZ12 fRIP GRID-seq mESC	3012	47458	0.0917
SUZ12 RADICL-seq 2FA mESC	17403	314152	0.1865
SUZ12 fRIP RADICL-seq 2FA mESC	14355	254532	0.2487
SUZ12 RADICL-seq NPM mESC	28444	608381	0.0453
SUZ12 fRIP RADICL-seq NPM mESC	10813	198167	0.1739
SUZ12 fRIP	29	194	0.7525
TAF15	76	359	0.4208
TARDBP	580	9031	0.1606
U2AF1	343	3644	0.3795
U2AF2	11	26	0.5698
WDR5 fRIP GRID-seq mESC	1	5	0.8182
WDR5 fRIP RADICL-seq 2FA mESC	4	419	0.5490

See next page

Supplementary Table S10 – continued from previous page

Protein	Cis-contacts number	Trans-contacts number	mRNA contacts ratio
WDR5 fRIP RADICL-seq NPM mESC	1	497	0.3656
WDR5 fRIP	733	9944	0.4998
YBX3	2	38	0.8326
ZC3H11A	82	637	0.4524
ZC3H8	877	9851	0.3657

Supplementary Table S11. cis-/trans- contacts ratio statistics for selected RNAs for K562 Red-C experiment.

RNA	RNA Type	Red-C cis-/trans-contacts ratio	Triads cis-/trans-contacts ratio
1840_HUVEC	vlinc	0.193256	0.130892
2125_K562	vlinc	0.177960	0.120279
2286_K562	vlinc	0.213551	0.170541
AC005208.1	lncRNA	0.168325	0.136384
AC012494.1	lncRNA	0.182849	0.064323
AC020687.1	lncRNA	0.290336	0.163065
AC097528.1	lncRNA	0.194500	0.031450
AL390334.1	pseudogene	0.252462	0.090108
AL445255.1	lncRNA	0.271939	0.073506
AL713998.1	lncRNA	0.074473	0.078996
B3GALT1-AS1	lncRNA	0.195343	0.098824
C22orf34	lncRNA	0.421999	0.474465
CCDC26	lncRNA	0.217844	0.394440
DSCR4	lncRNA	0.512160	0.730741
FTX	lncRNA	0.085945	0.089114
KCNQ1OT1	lncRNA	0.293347	0.352229
LINC00355	lncRNA	0.273391	0.023474

See next page

Supplementary Table S11 – continued from previous page

RNA	RNA Type	Red-C cis-/trans-contacts ratio	Triads cis-/trans-contacts ratio
LINC00470	lncRNA	0.231691	0.160800
LINC02476	lncRNA	0.243996	0.458085
MIR4435-2HG	lncRNA	0.214966	0.153130
MIR548XHG	lncRNA	0.431875	0.222322
RMRP_2	ribozyme	0.037462	0.033039
RPPH1	ribozyme	0.047262	0.020858
U2_728	snRNA	0.055386	0.057798
X_13_10942_a_hg38	Xrna	0.036283	0.011467
X_2_7832_a_hg38	Xrna	0.198941	0.124180
X_4_12686_b_hg38	Xrna	0.205287	0.082588

Supplementary Table S12. Triads and ChIP-seq composition by ChromHMM annotation of DNA loci for all proteins split by function. *cnt* – number of observed ChIP-seq loci in a state; *tri* – number of triads; *Z* – z-score of $\frac{tri}{cnt}$ distribution over all ChromHMM states

State			All proteins			Remodelling		Tr.Reg		RNA proc.	
	$\frac{cnt}{10^4}$	%	$\frac{tri}{10^3}$	%	Z	$\frac{tri}{10^3}$	Z	$\frac{tri}{10^3}$	Z	$\frac{tri}{10^3}$	Z
Promoter	56.51	23.58	848.70	34.69	-0.22	806.37	-0.23	24.10	0.38	18.23	0.54
Enhancer	108.77	45.39	455.95	18.63	-0.64	398.61	-0.63	32.86	-0.35	24.48	-0.24
Transcribed	40.59	16.94	191.08	7.81	-0.62	158.96	-0.62	14.47	-0.03	17.64	1.45
Insulator	6.93	2.89	90.35	3.69	-0.30	88.84	-0.28	0.84	-1.42	0.67	-1.28
PRC_Repressed	7.95	3.32	582.11	23.79	2.01	578.35	2.01	2.59	-0.21	1.17	-0.87
HetChrom_CNV	18.90	7.89	278.60	11.39	-0.23	260.74	-0.24	12.08	1.63	5.78	0.41
TOTAL	239.66	100.00	2446.79	100.00	-0.41	2291.87	-0.41	86.94	0.01	67.98	0.23

Supplementary Table S13. Triads and RD-contacts DNA parts composition by ChromHMM annotation of DNA loci for all proteins split by function. *cnt* – number of observed DNA-parts in a state; *tri* – number of triads; *Z* – z-score of $\frac{tri}{cnt}$ distribution over all ChromHMM states

**State**	$\frac{cnt}{10^6}$	%	**All proteins**			**Remodelling**		**Tr.Reg**		**RNA proc.**	
			$\frac{tri}{10^3}$	%	Z	$\frac{tri}{10^3}$	Z	$\frac{tri}{10^3}$	Z	$\frac{tri}{1000}$	Z
Promoter	6.36	2.78	848.70	34.69	1.98	806.37	1.97	24.10	1.78	18.23	1.76
Enhancer	16.48	7.20	455.95	18.63	-0.23	398.61	-0.27	32.86	0.56	24.48	0.51
Transcribed	20.31	8.88	191.08	7.81	-0.61	158.96	-0.63	14.47	-0.31	17.64	-0.05
Insulator	2.72	1.19	90.35	3.69	-0.12	88.84	-0.09	0.84	-0.58	0.67	-0.62
PRC_Repressed	21.64	9.46	582.11	23.79	-0.25	578.35	-0.22	2.59	-0.71	1.17	-0.79
HetChrom_CNV	161.30	70.49	278.60	11.39	-0.77	260.74	-0.77	12.08	-0.74	5.78	-0.81
TOTAL	228.81	100.00	2446.79	100.00	-0.58	2291.87	-0.58	86.94	-0.54	67.98	-0.57

Supplementary Table S14. Triads and ChIP-seq composition by SPIN annotation of DNA loci for all proteins split by function. *cnt* – number of observed ChIP-seq loci in a state; *tri* – number of triads; *Z* – z-score of $\frac{tri}{cnt}$ distribution over all SPIN states

**State**	$\frac{cnt}{10^4}$	%	**All proteins**			**Remodelling**		**Tr.Reg**		**RNA proc.**	
			$\frac{tri}{10^3}$	%	Z	$\frac{tri}{10^3}$	Z	$\frac{tri}{10^3}$	Z	$\frac{tri}{10^3}$	Z
A_compartment	21.58	53.87	235.24	54.56	0.90	153.07	0.99	42.79	0.75	39.38	0.69
B_compartment	1.68	4.19	13.09	3.04	-0.40	9.57	-0.14	2.08	-0.72	1.44	-0.54
Lamina	0.62	1.56	3.76	0.87	-1.14	2.53	-1.45	0.96	-0.13	0.27	-1.09
Lamina_like	0.32	0.79	2.26	0.52	-0.69	1.58	-0.73	0.37	-0.90	0.32	-0.36
Near_Lm	4.73	11.81	39.60	9.18	-0.16	28.85	0.18	6.06	-0.64	4.70	-0.37
Speckle	11.13	27.79	137.18	31.82	1.49	81.27	1.15	27.12	1.64	28.79	1.67
TOTAL	40.07	100.00	431.13	100.00	0.84	276.87	0.84	79.37	0.74	74.90	0.75

Supplementary Table S15. Triads and and RD-contacts DNA parts composition by SPIN annotation of DNA loci for all proteins split by function. *cnt* – number of of observed DNA-parts in a state; *tri* – number of triads; *Z* – z-score of $\frac{tri}{cnt}$ distribution over all SPIN states

**State**	$\frac{cnt}{10^5}$	%	**All proteins**			**Remodelling**		**Tr.Reg**		**RNA proc.**	
			$\frac{tri}{10^3}$	%	Z	$\frac{tri}{10^3}$	Z	$\frac{tri}{10^3}$	Z	$\frac{tri}{10^3}$	Z
A_compartment	205.61	37.63	235.24	54.56	0.22	153.07	0.28	42.79	0.18	39.38	0.11
B_compartment	49.58	9.08	13.09	3.04	-0.50	9.57	-0.49	2.08	-0.50	1.44	-0.51
Lamina	100.68	18.43	3.76	0.87	-0.68	2.53	-0.72	0.96	-0.63	0.27	-0.61
Lamina_like	8.91	1.63	2.26	0.52	-0.50	1.58	-0.51	0.37	-0.50	0.32	-0.48
Near_Lm	139.20	25.48	39.60	9.18	-0.48	28.85	-0.47	6.06	-0.49	4.70	-0.49
Speckle	42.33	7.75	137.18	31.82	1.94	81.27	1.92	27.12	1.95	28.79	1.97
TOTAL	546.32	100.00	431.13	100.00	-0.07	276.87	-0.05	79.37	-0.08	74.90	-0.10

Supplementary Table S16. Triads and and RD-contacts composition by RNA biotypes for all proteins split by function. *cnt* – number of of observed RNA of a given biotype; *tri* – number of triads; *Z* – z-score of $\frac{tri}{cnt}$ distribution over all RNA biotypes

Biotypes	$\frac{cnt}{10^4}$	%	All proteins			Remodelling		Tr.Reg		RNA proc.	
			$\frac{tri}{10^3}$	%	Z	$\frac{tri}{10^3}$	Z	$\frac{tri}{10^3}$	Z	$\frac{tri}{10^3}$	Z
mRNA	87.14	66.56	471.91	19.61	-0.45	409.31	-0.44	32.69	-0.60	29.90	-0.49
rRNA	0.23	0.18	853.58	35.47	2.62	850.18	2.62	3.19	0.44	0.21	-0.45
snRNA	0.39	0.30	216.48	8.99	0.00	186.63	-0.05	15.25	2.39	14.60	2.50
snoRNA	0.38	0.29	217.21	9.03	0.02	206.78	0.01	5.25	0.44	5.19	0.58
lncRNA	22.67	17.32	113.43	4.71	-0.45	96.34	-0.44	9.13	-0.60	7.97	-0.49
X-RNA	11.52	8.80	431.17	17.92	-0.42	414.85	-0.41	8.57	-0.57	7.75	-0.46
miRNA	0.76	0.58	20.27	0.84	-0.43	20.23	-0.42	0.04	-0.63	0.00	-0.52
pseudo	6.08	4.65	20.16	0.84	-0.45	11.78	-0.44	2.81	-0.60	2.42	-0.48
other	1.74	1.33	62.49	2.60	-0.43	44.41	-0.42	8.08	-0.27	6.87	-0.19
TOTAL	130.92	100.00	2406.70	100.00	-0.44	2240.51	-0.43	85.01	-0.58	74.90	-0.47

Supplementary Table S17. -log10(*p*-values) for the real number of triads on the background distribution of the number of triads obtained on simulated data. The background distribution for triads number was fit using a gamma distribution, and the *p*-value defined as the probability of obtaining the real number of triads for protein or a value greater from the fit distribution.

Triads constructed on	*p*-value of simulation	adjusted *p*-value
CBP Red-C fRIP	141.331	140.84
CBX3 Red-C fRIP	102.742	102.412
CHD1 Red-C fRIP	102.54	102.224
CHD4 Red-C fRIP	162.695	162.181
CHD7 Red-C fRIP	125.304	124.854
CTCF Red-C fRIP	180.472	179.91
DNMT1 Red-C fRIP	18.547	18.426
EWSR1 Red-C eCLIP	104.636	104.291
EZH2 GRID eCLIP	296.277	295.631
EZH2 RADICL 2FA eCLIP	296.277	295.631
EZH2 RADICL NPM eCLIP	296.277	295.631
EZH2 Red-C fRIP	63.279	63.054
FUS Red-C eCLIP	3.27	3.248

See next page

Supplementary Table S17 – continued from previous page

**Triads constructed on**	**-log10(p-values) of simulation**	**-log10(adjusted p-value)**
GTF2F1 Red-C eCLIP	115.802	115.407
HDAC1 Red-C fRIP	120.077	119.647
HLTF Red-C eCLIP	177.046	176.509
HNRNPC Red-C eCLIP	8.816	8.756
HNRNPH Red-C fRIP	7.009	6.964
HNRNPK GRID eCLIP	52.601	52.421
HNRNPK RADICL2FA eCLIP	296.277	295.631
HNRNPK RADICL NPM eCLIP	296.277	295.631
HNRNPK Red-C eCLIP	54.8	54.61
HNRNPL Red-C eCLIP	2.346	2.332
HNRNPUL1 Red-C eCLIP	110.168	109.807
ILF3 Red-C eCLIP	33.853	33.704
KHSRP Red-C eCLIP	3.504	3.475
LARP7 Red-C eCLIP	119.872	119.46
LSD1 Red-C fRIP	8.851	8.783
MORC2 Red-C eCLIP	66.157	65.896
NONO Red-C eCLIP	27.661	27.521
NUP98 Red-C fRIP	296.277	295.631
PCAF Red-C fRIP	62.037	61.824
PCBP1 Red-C eCLIP	111.221	110.844
PHF8 Red-C fRIP	296.277	295.631
PTBP1 Red-C eCLIP	296.277	295.631
RBBP5 Red-C fRIP	296.277	295.631
RBFOX2 Red-C eCLIP	56.393	56.192
RBM15 Red-C eCLIP	40.249	40.09
RBM22 Red-C eCLIP	90.91	90.636

See next page

Supplementary Table S17 – continued from previous page

Triads constructed on	-log10(p-values) of simulation	-log10(adjusted p-value)
SAFB2 Red-C eCLIP	8.024	7.972
SAFB Red-C eCLIP	12.453	12.36
SRSF1 Red-C eCLIP	12.768	12.666
SRSF7 Red-C eCLIP	19.857	19.727
SRSF9 Red-C eCLIP	9.63	9.545
SUZ12 GRID eCLIP	296.277	295.631
SUZ12 GRID fRIP	296.277	295.631
SUZ12 RADICL 2FA eCLIP	296.277	295.631
SUZ12 RADICL 2FA fRIP	296.277	295.631
SUZ12 RADICL NPM eCLIP	99.435	99.134
SUZ12 RADICL NPM fRIP	192.606	191.989
SUZ12 Red-C fRIP	3.823	3.787
TAF15 Red-C eCLIP	65.584	65.336
TARDBP Red-C eCLIP	296.277	295.631
U2AF1 Red-C eCLIP	98.987	98.7
U2AF2 Red-C eCLIP	9.369	9.292
WDR5 GRID fRIP	1.653	1.653
WDR5 RADICL 2FA fRIP	52.543	52.374
WDR5 RADICL NPM fRIP	64.79	64.554
WDR5 Red-C fRIP	14.914	14.802
YBX3 Red-C eCLIP	1.811	1.804
ZC3H11A Red-C eCLIP	126.45	125.98
ZC3H8 Red-C eCLIP	191.491	190.903

Supplementary Table S18. Results for the association tests: χ^2- goodness-of-fit of ChromHMM annotation of DNA-parts and Fisher's exact test for association of PR- and PD-contacts data of protein with RD-contacts.

**Protein**	**q-value(χ^2)**	**q-value(Fisher)**
CBP	$5.10 * 10^{-18}$	$7.86 * 10^{-6}$
CBX3	$2.36 * 10^{-61}$	$7.41 * 10^{-5}$
CHD1	$3.14 * 10^{-214}$	$1.25 * 10^{-1}$
CHD4	$8.27 * 10^{-57}$	$1.01 * 10^{-3}$
CHD7	$3.68 * 10^{-36}$	$3.61 * 10^{-4}$
CTCF	0	$4.45 * 10^{-1}$
DNMT1	$1.50 * 10^{-6}$	$9.62 * 10^{-5}$
EWSR1	$3.68 * 10^{-36}$	$9.62 * 10^{-5}$
EZH2	$5.30 * 10^{-40}$	0.001060326675
FUS	$1.37 * 10^{-31}$	$7.87 * 10^{-4}$
GTF2F1	0	$3.14 * 10^{-4}$
HDAC1	$1.56 * 10^{-97}$	0.01977
HLTF	$8.24 * 10^{-19}$	0.000173
HNRNPC	$7.74 * 10^{-5}$	$3.59 * 10^{-2}$
HNRNPH	$5.87 * 10^{-6}$	$1.73 * 10^{-8}$
HNRNPK	$3.23 * 10^{-303}$	$6.74 * 10^{-2}$
HNRNPL	$4.09 * 10^{-9}$	0.0001024
HNRNPUL1	$7.20 * 10^{-94}$	$9.62 * 10^{-5}$
ILF3	$7.37 * 10^{-5}$	0.003031
KHSRP	0.78792	0.0004923
LARP7	$1.30 * 10^{-118}$	$9.46 * 10^{-2}$
LSD1	0	0.4440061483
MORC2	$1.33 * 10^{-105}$	$5.50 * 10^{-3}$
NONO	$2.65 * 10^{-9}$	$7.87 * 10^{-4}$
NUP98	$2.01 * 10^{-86}$	0.016093
PCAF	$5.71 * 10^{-14}$	0.0007874

See next page

Supplementary Table S18 – continued from previous page

Protein	q-value(χ^2)	q-value(Fisher)
PCBP1	$6.08 * 10^{-115}$	$9.50 * 10^{-2}$
PHF8	0	0.36820
PTBP1	$1.28 * 10^{-91}$	0.01575443137
RBBP5	$1.11 * 10^{-188}$	$3.68 * 10^{-1}$
RBFOX2	$1.95 * 10^{-60}$	0.001525
RBM15	0.000103582	0.0004245
RBM22	0	$1.25 * 10^{-1}$
SAFB	$2.65 * 10^{-31}$	$5.02 * 10^{-4}$
SAFB2	0.0002162	$4.78 * 10^{-4}$
SRSF1	0.071047	$1.24 * 10^{-6}$
SRSF7	0.210818	$1.76 * 10^{-5}$
SRSF9	0.039051	1.00
SUZ12	$7.62 * 10^{-15}$	$3.99 * 10^{-4}$
TAF15	$1.49 * 10^{-41}$	$7.86 * 10^{-6}$
TARDBP	$2.41 * 10^{-93}$	$9.50 * 10^{-2}$
U2AF1	$2.55 * 10^{-48}$	0.011595
U2AF2	0.000608817	$1.73 * 10^{-8}$
WDR5	$9.06 * 10^{-221}$	0.21304
YBX3	0.68586	0.0016564
ZC3H11A	$1.47 * 10^{-63}$	$3.03 * 10^{-5}$
ZC3H8	$1.44 * 10^{-98}$	$1.25 * 10^{-1}$

Supplementary Table S19. Results for the association test: χ^2- goodness-of-fit of SPIN annotation of DNA-parts of triads and proteins ChIP-seq loci.

Protein	p-value(χ^2)	q-value(χ^2)
CBP	$2.51 * 10^{-34}$	$6.95 * 10^{-34}$
CBX3	$1.06 * 10^{-22}$	$2.07 * 10^{-22}$

See next page

**Protein**	**p-value(χ^2)**	**q-value(χ^2)**
CHD1	$1.72 * 10^{-84}$	$1.01 * 10^{-83}$
CHD4	$1.00 * 10^{-20}$	$1.89 * 10^{-20}$
CHD7	$6.01 * 10^{-32}$	$1.57 * 10^{-31}$
CTCF	0	0
DNMT1	$4.02 * 10^{-14}$	$6.99 * 10^{-14}$
EWSR1	$2.26 * 10^{-74}$	$1.06 * 10^{-73}$
EZH2	$1.13 * 10^{-25}$	$2.52 * 10^{-25}$
FUS	$2.62 * 10^{-6}$	$3.84 * 10^{-6}$
GTF2F1	$1.46 * 10^{-27}$	$3.43 * 10^{-27}$
HDAC1	$1.29 * 10^{-28}$	$3.19 * 10^{-28}$
HLTF	$1.63 * 10^{-5}$	$2.25 * 10^{-5}$
HNRNPC	$2.93 * 10^{-2}$	$3.36 * 10^{-2}$
HNRNPH	$3.80 * 10^{-3}$	$4.71 * 10^{-3}$
HNRNPK	$1.23 * 10^{-240}$	$2.90 * 10^{-239}$
HNRNPL	$1.52 * 10^{-1}$	$1.59 * 10^{-1}$
HNRNPUL1	$1.03 * 10^{-18}$	$1.87 * 10^{-18}$
ILF3	$7.11 * 10^{-2}$	$7.77 * 10^{-2}$
KHSRP	$1.43 * 10^{-2}$	$1.68 * 10^{-2}$
LARP7	$1.33 * 10^{-69}$	$5.20 * 10^{-69}$
LSD1	$3.12 * 10^{-122}$	$2.10 * 10^{-121}$
MORC2	$3.04 * 10^{-62}$	$1.10 * 10^{-61}$
NONO	$2.65 * 10^{-54}$	$8.31 * 10^{-54}$
NUP98	$1.49 * 10^{-81}$	$7.78 * 10^{-81}$
PCAF	$1.76 * 10^{-7}$	$2.66 * 10^{-7}$
PCBP1	$8.67 * 10^{-186}$	$1.02 * 10^{-184}$
PHF8	$1.12 * 10^{-153}$	$8.80 * 10^{-153}$

See next page

Supplementary Table S19 – continued from previous page

**Protein**	**p-value(χ^2)**	**q-value(χ^2)**
PTBP1	$2.21 * 10^{-23}$	$4.52 * 10^{-23}$
RBBP5	$1.43 * 10^{-185}$	$1.35 * 10^{-184}$
RBFOX2	$1.93 * 10^{-13}$	$3.23 * 10^{-13}$
RBM15	$5.92 * 10^{-10}$	$9.28 * 10^{-10}$
RBM22	$3.31 * 10^{-11}$	$5.36 * 10^{-11}$
SAFB	$5.37 * 10^{-3}$	$6.47 * 10^{-3}$
SAFB2	$8.39 * 10^{-2}$	$8.96 * 10^{-2}$
SRSF1	$8.30 * 10^{-5}$	$1.05 * 10^{-4}$
SRSF7	$2.93 * 10^{-1}$	$3.00 * 10^{-1}$
SRSF9	$3.90 * 10^{-5}$	$5.09 * 10^{-5}$
SUZ12	$1.64 * 10^{-24}$	$3.50 * 10^{-24}$
TAF15	$3.05 * 10^{-1}$	$3.05 * 10^{-1}$
TARDBP	$1.66 * 10^{-54}$	$5.59 * 10^{-54}$
U2AF1	$3.09 * 10^{-48}$	$9.08 * 10^{-48}$
U2AF2	$2.18 * 10^{-5}$	$2.92 * 10^{-5}$
WDR5	$1.99 * 10^{-210}$	$3.11 * 10^{-209}$
YBX3	$4.18 * 10^{-2}$	$4.67 * 10^{-2}$
ZC3H11A	$1.59 * 10^{-5}$	$2.25 * 10^{-5}$
ZC3H8	$6.35 * 10^{-70}$	$2.71 * 10^{-69}$

Supplementary Table S20. RNA orthologs found by ortho2align (selected as bidirectional best hit)

**Human RNA**	**Mouse RNA**	**In triads with**	**Human biotype**	**Mouse biotype**
U79	Gas5	EZH2	CDBox	processed transcript
	Gm50452	EZH2	CDBox	snoRNA
RPPH1	Rpph1	EZH2, hnRNPK	ribozyme	ribozyme
SNORD58B	Gm23301	EZH2, hnRNPK	snoRNA	snoRNA

See next page

Supplementary Table S20 – continued from previous page

Human RNA	Mouse RNA	In triads with	Human biotype	Mouse biotype
RNU4-2	Gm24265	EZH2, SUZ12	snRNA	snRNA
C19orf48	Gm26247	EZH2	pseudogene	snoRNA
	Snord88a	EZH2	pseudogene	snoRNA
	Gm23991	EZH2	pseudogene	snoRNA
DLEU2	Dleu2	hnRNPK	lncRNA	processed transcript
RNF213-AS1	A930037H05Rik	SUZ12	lncRNA	processed transcript
SNORD5	Gm24299	SUZ12	snoRNA	snoRNA

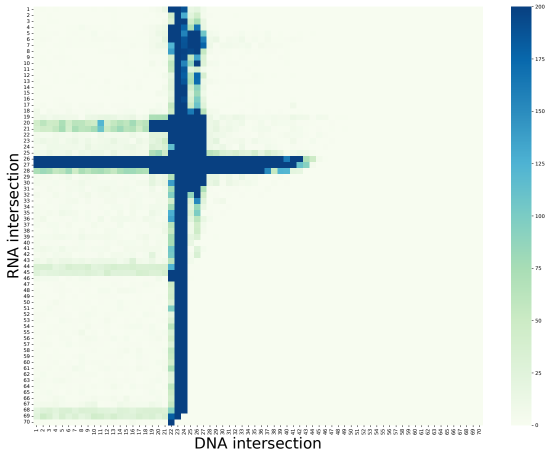

Supplementary Figure S1. Threshold selection for triads construction filtering using size of intersection between peaks and correspondent part of an RNA-DNA contact.

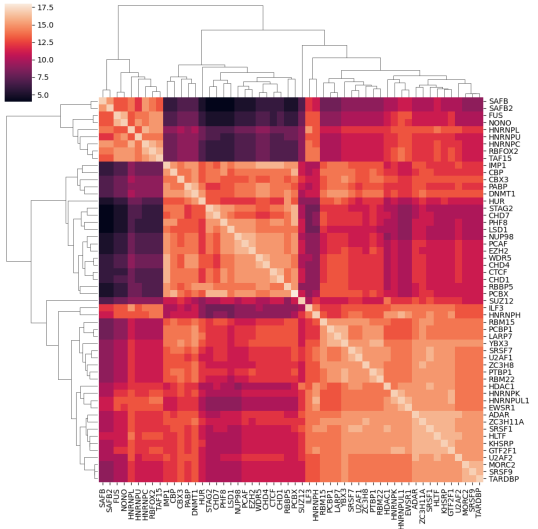

Supplementary Figure S2. Protein clusterization based on their "related" proteins

CBP Red-C fRIP-seq K562 | CBX3 Red-C fRIP-seq K562 | CHD1 Red-C fRIP-seq K562 | CHD4 Red-C fRIP-seq K562

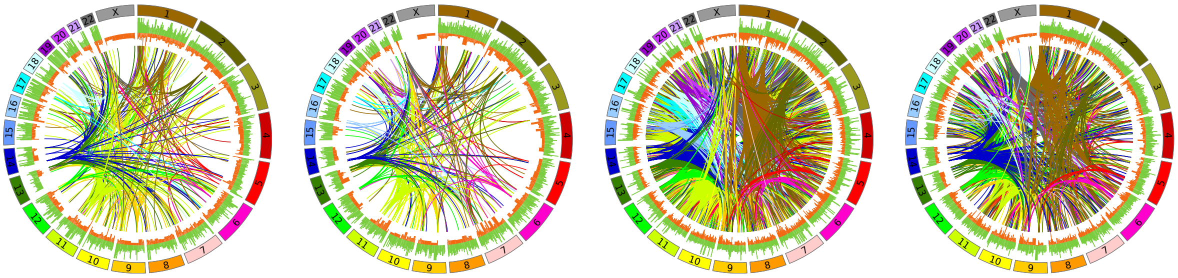

CHD7 Red-C fRIP-seq K562

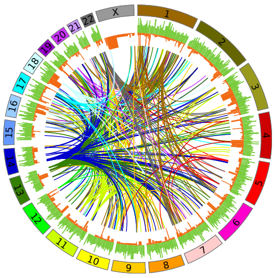

CTCF Red-C fRIP-seq K562

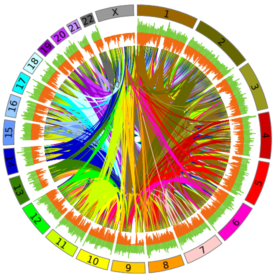

DNMT1 Red-C fRIP-seq K562

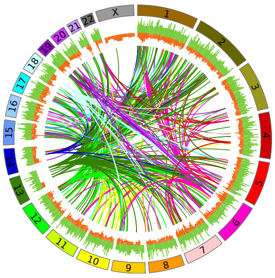

EWSR1 Red-C eCLIP-seq K562

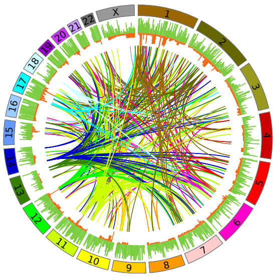

EZH2 GRID-seq eCLIP-seq mESC

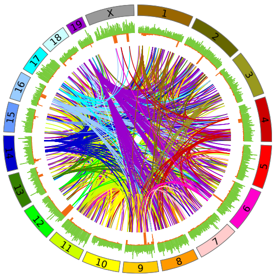

EZH2 RADICL-seq 2FA eCLIP-seq mESC

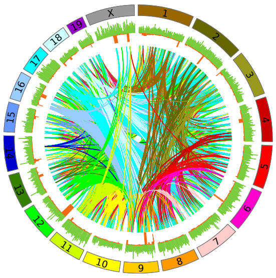

EZH2 RADICL-seq NPM eCLIP-seq mESC

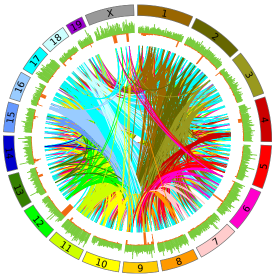

EZH Red-C fRIP-seq K562

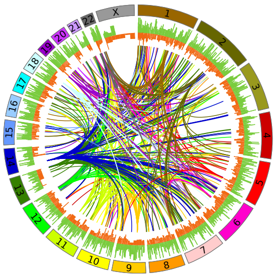

FUS Red-C eCLIP-seq K562

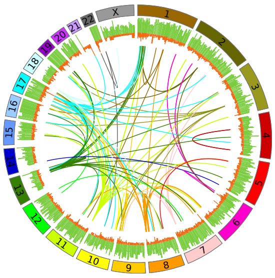

GTF2F1 Red-C eCLIP-seq K562

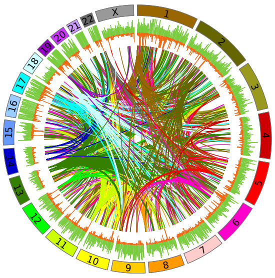

HDAC1 Red-C fRIP-seq K562

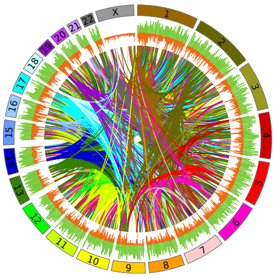

HLTF Red-C eCLIP-seq K562

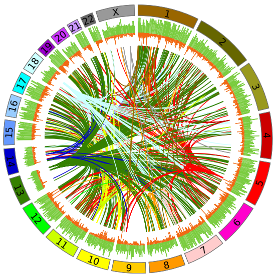

hnRNPC Red-C eCLIP-seq K562

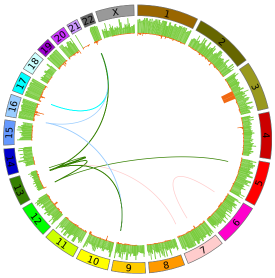

hnRNPH Red-C fRIP-seq K562

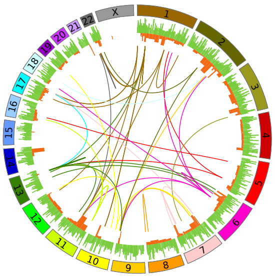

hnRNPK GRID-seq eCLIP-seq mESC

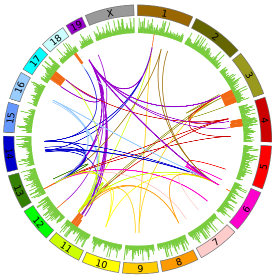

hnRNPK RADICL-seq 2FA eCLIP-seq mESC

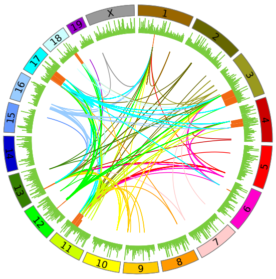

hnRNPK RADICL-seq NPM eCLIP-seq K562

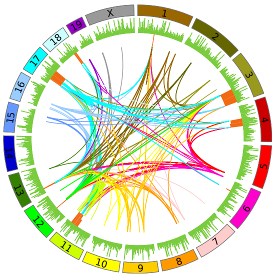

hnRNPK Red-C eCLIP-seq

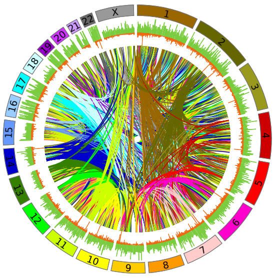

hnRNPL Red-C eCLIP-seq K562

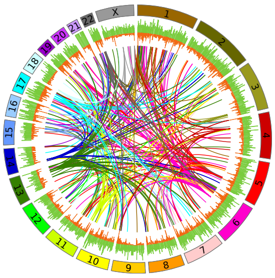

hnRNPUL1 Red-C eCLIP-seq K562

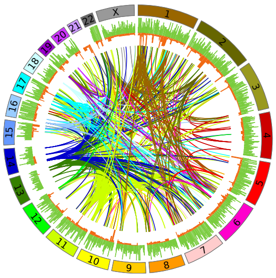

ILF3 Red-C eCLIP-seq K562

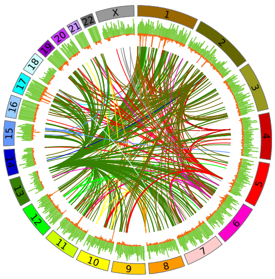

KHSRP Red-C eCLIP-seq K562

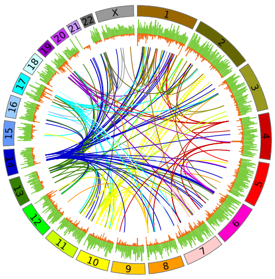

LARP7 Red-C eCLIP-seq K562

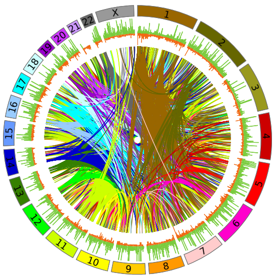

LSD1 Red-C fRIP-seq K562

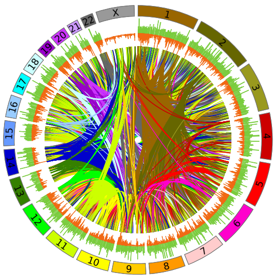

MORC2 Red-C eCLIP-seq K562

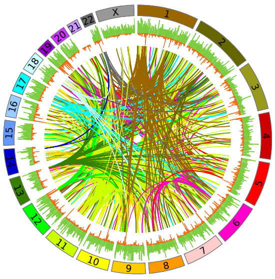

NONO Red-C eCLIP-seq K562

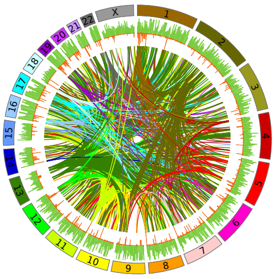

NUP98 Red-C fRIP-seq K562

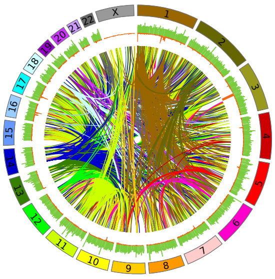

PCAF Red-C fRIP-seq K562

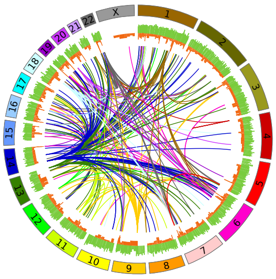

PCBP1 Red-C eCLIP-seq K562

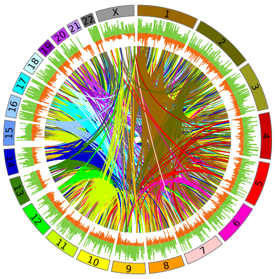

PHF8 Red-C fRIP-seq K562

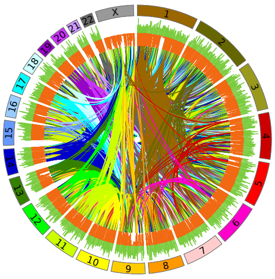

PTBP1 Red-C eCLIP-seq K562

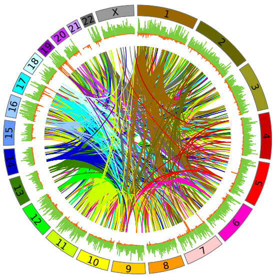

RBBP5 Red-C fRIP-seq K562

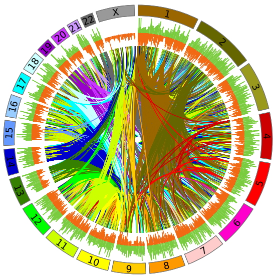

RBFOX2 Red-C
eCLIP-seq K562

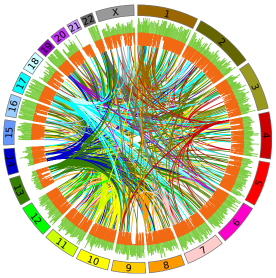

RBM15 Red-C
eCLIP-seq K562

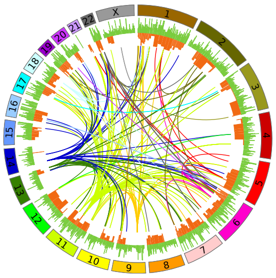

RBM22 Red-C
eCLIP-seq K562

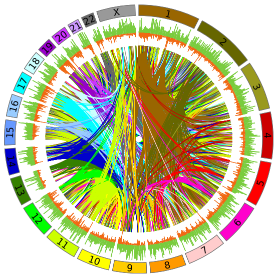

SAFB Red-C
eCLIP-seq K562

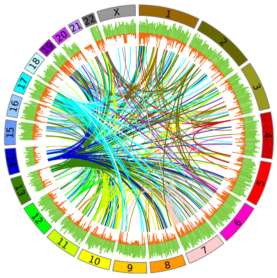

SAFB2 Red-C
eCLIP-seq K562

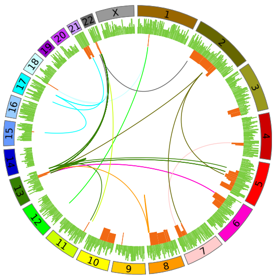

SRSF1 Red-C
eCLIP-seq K562

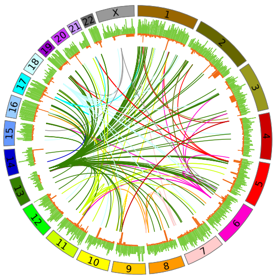

SRSF7 Red-C
eCLIP-seq K562

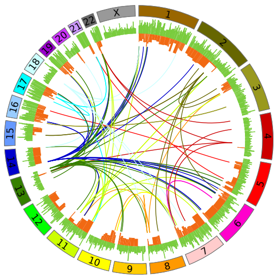

SRSF9 Red-C
eCLIP-seq K562

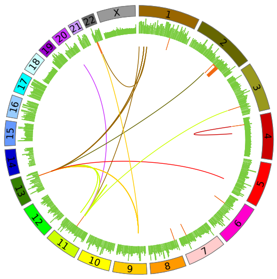

SUZ12 GRID-seq
fRIP-seq mESC

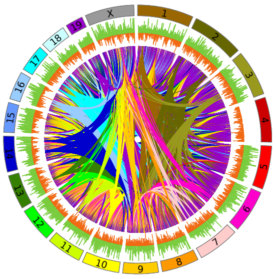

SUZ12 GRID-seq
eCLIP-seq mESC

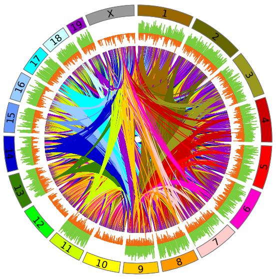

SUZ12 RADICL-seq
2FA fRIP-seq mESC

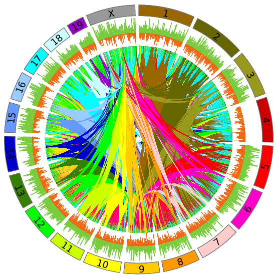

SUZ12 RADICL-seq
2FA eCLIP-seq mESC

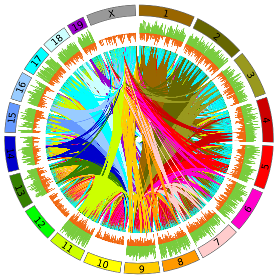

SUZ12 RADICL-seq
NPM fRIP-seq mESC

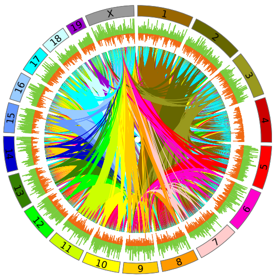

SUZ12 RADICL-seq
NPM eCLIP-seq
mESC

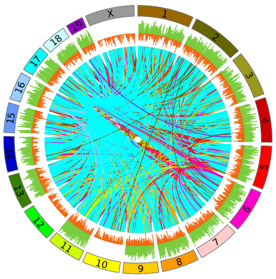

SUZ12 Red-C
fRIP-seq K562

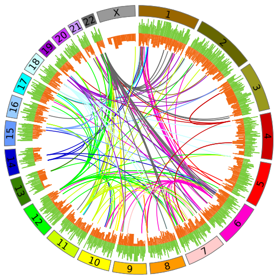

TAF15 Red-C
eCLIP-seq K562

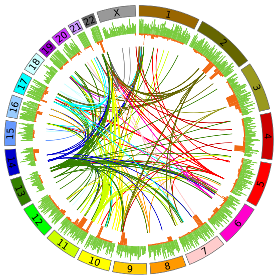

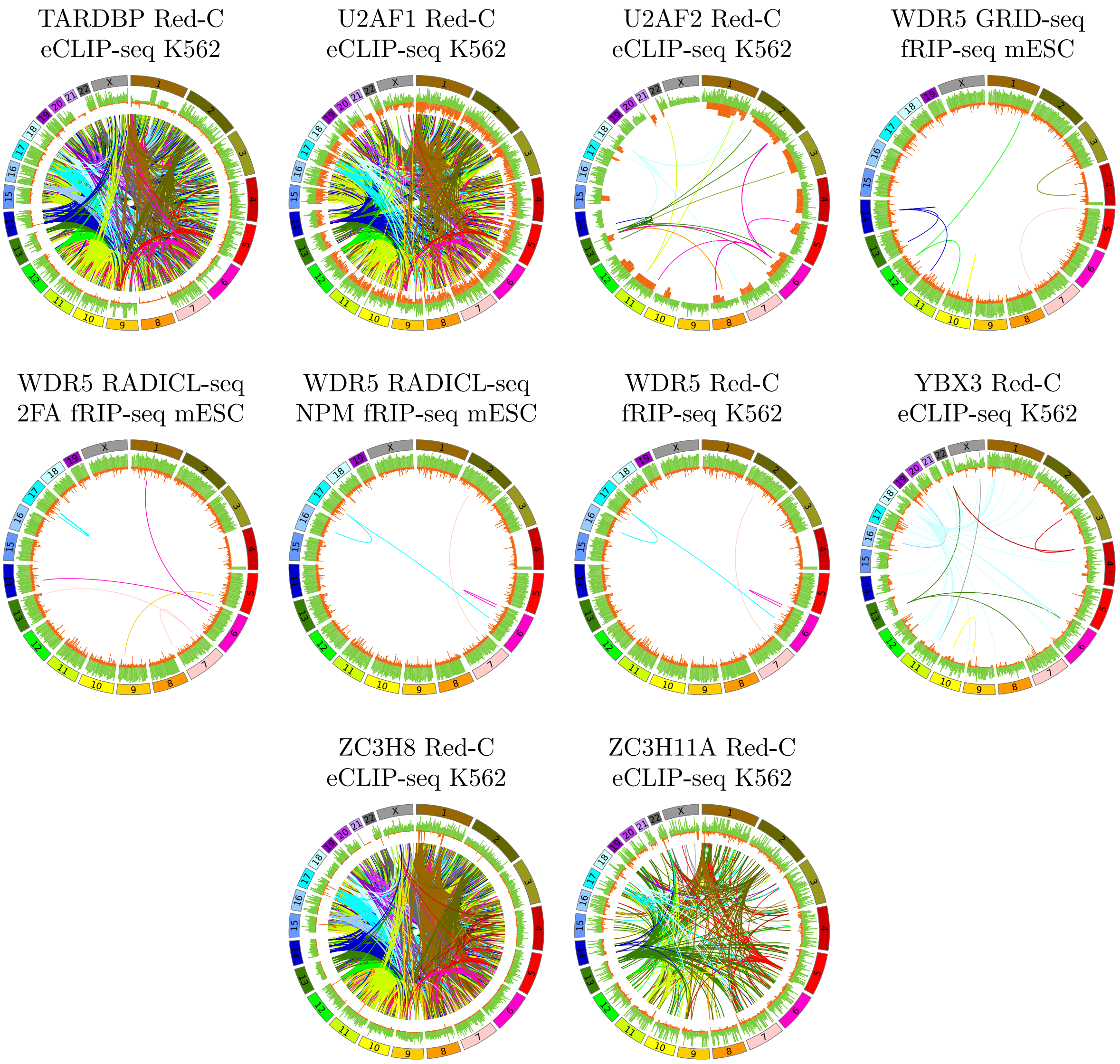

Supplementary Figure S3. Circos diagrams for constructed protein mediated RNA-DNA interactions, or triads. Legend for diagrams is shown in the main text.

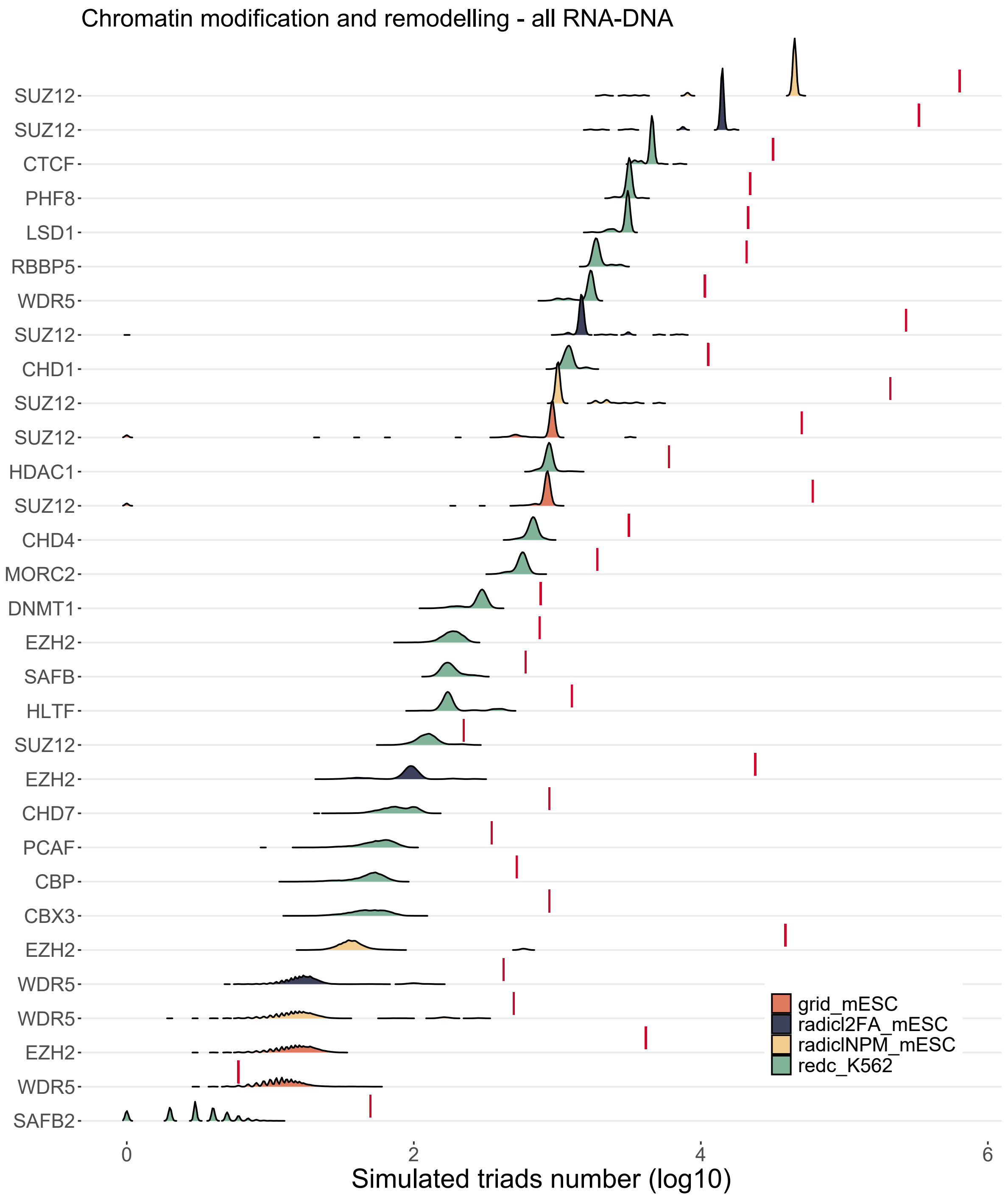

Supplementary Figure S4. Ridgeline plot for number of simulated triads for chromatin remodelling proteins.

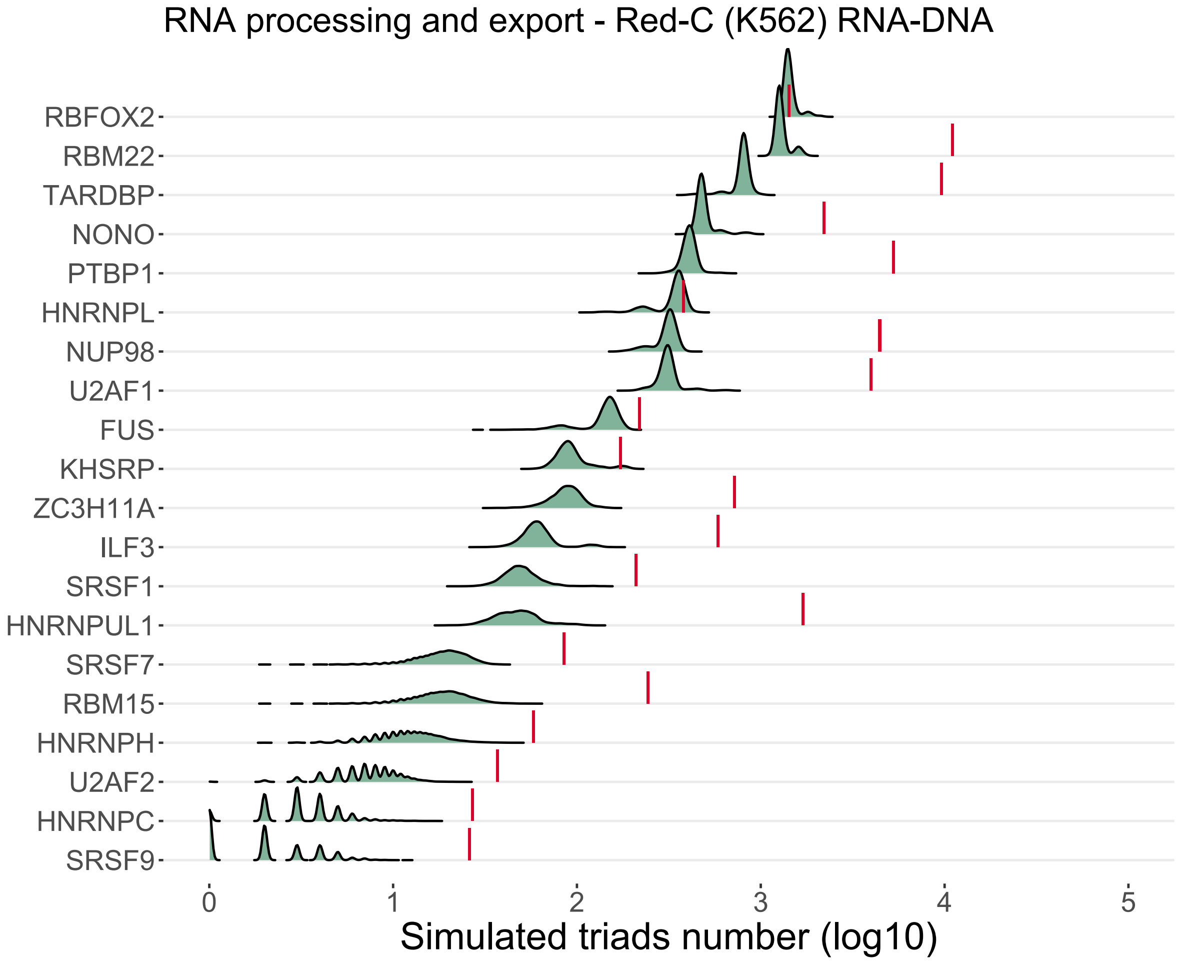

Supplementary Figure S5. Ridgeline plot for number of simulated triads for RNA processing and export proteins.

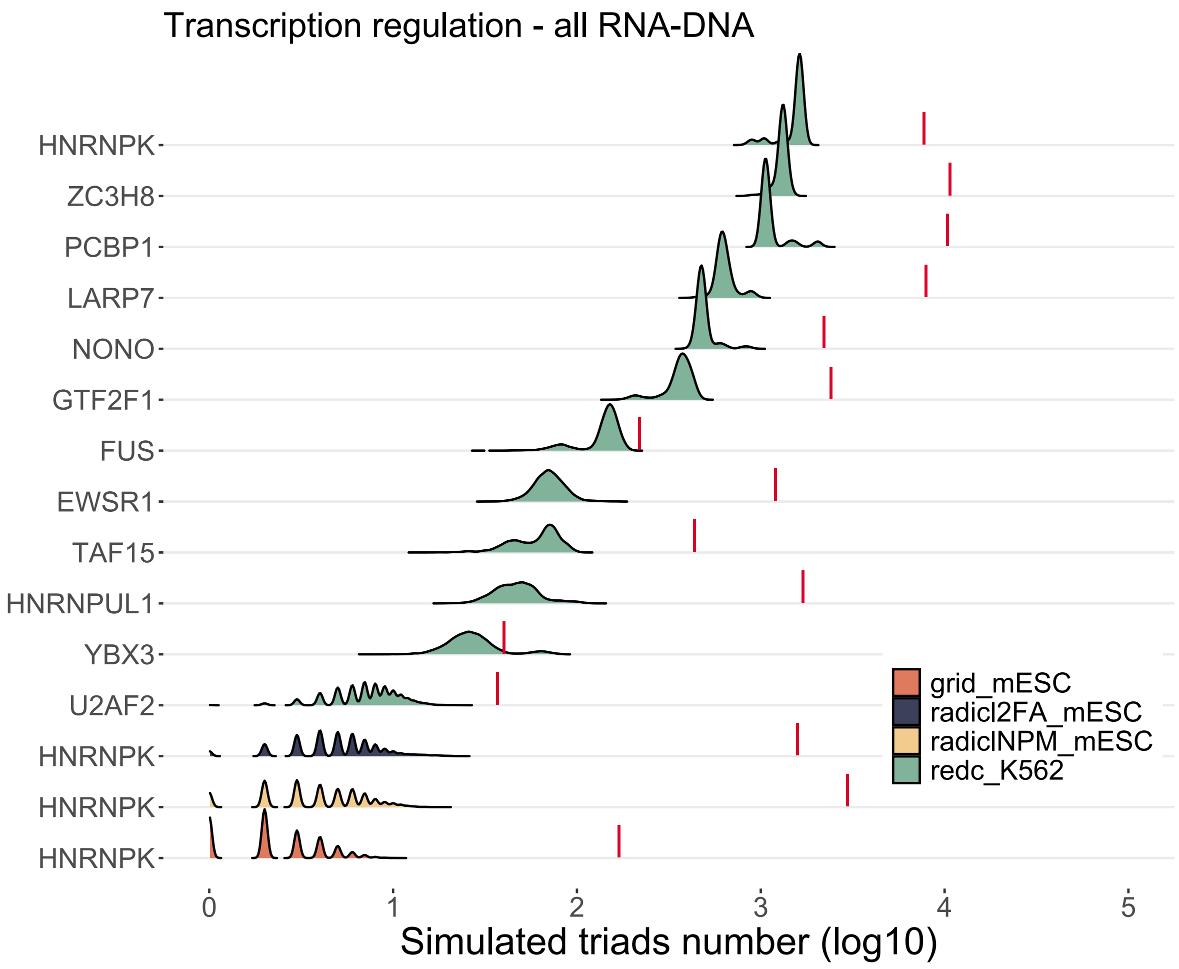

Supplementary Figure S6. Ridgeline plot for number of simulated triads for transcription regulating proteins.

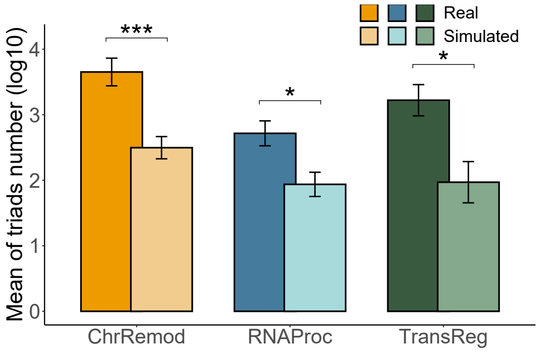

Supplementary Figure S7. Barplots displaying the number of simulated triads for each functional group of proteins.

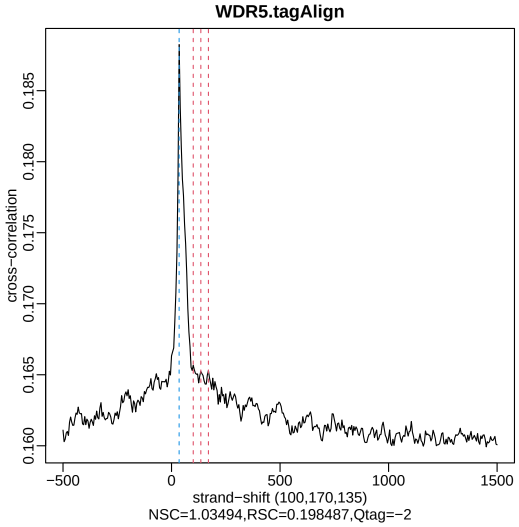

Supplementary Figure S8. Strand cross-correlation for WDR5 ChIP-seq.

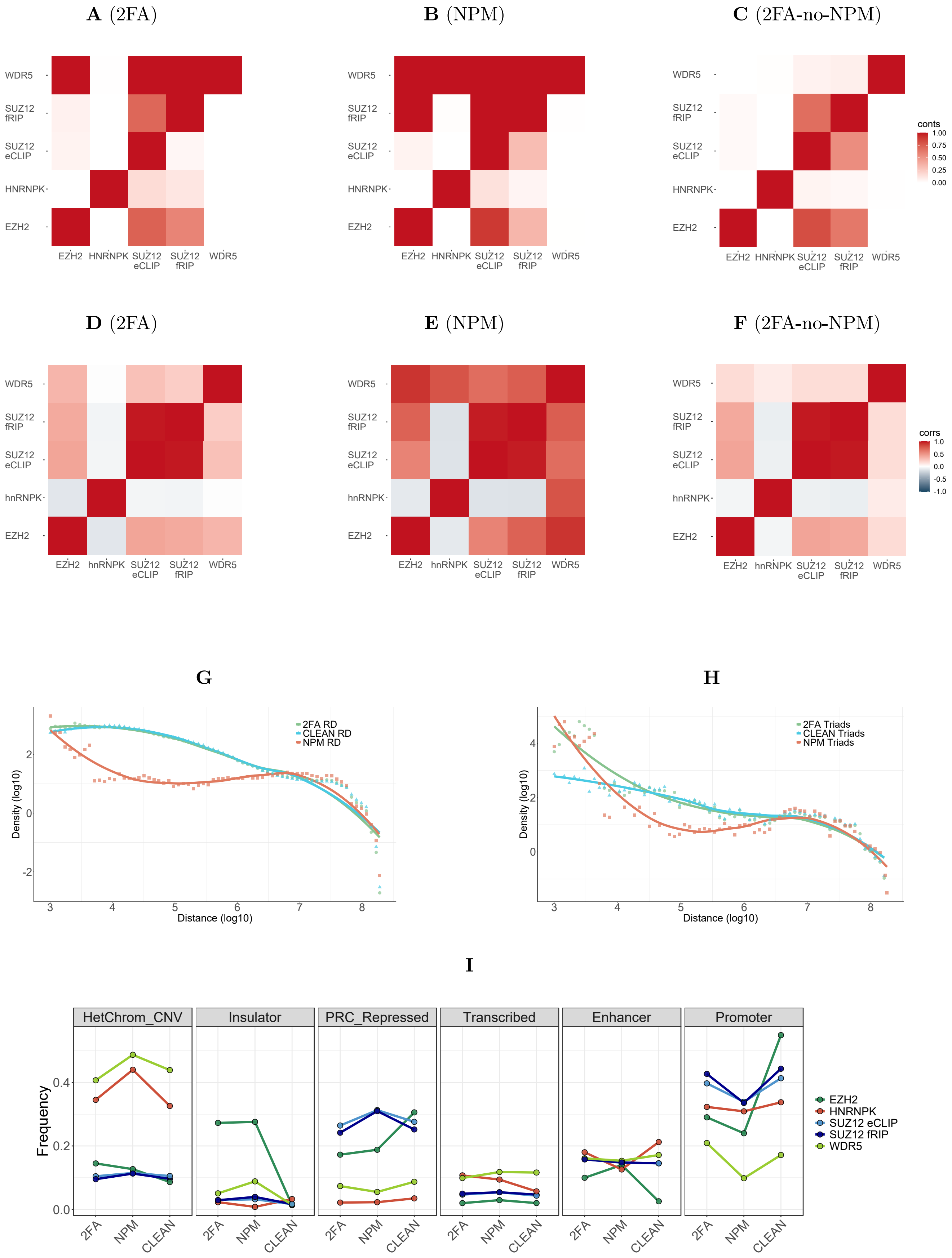
A (2FA)
B (NPM)
C (2FA-no-NPM)
WDR5
SUZ12 fRIP
SUZ12 eCLIP
HNRNPK
EZH2
conts
1.00
0.75
0.50
0.25
0.00
D (2FA)
E (NPM)
F (2FA-no-NPM)
hnRNPK
corrs
1.0
0.5
0.0
-0.5
-1.0
G
H
2FA RD
CLEAN RD
NPM RD
2FA Triads
CLEAN Triads
NPM Triads
Density (log10)
Distance (log10)
I
HetChrom_CNV
Insulator
PRC_Repressed
Transcribed
Enhancer
Promoter
Frequency
2FA
NPM
CLEAN
EZH2
HNRNPK
SUZ12 eCLIP
SUZ12 fRIP
WDR5

Supplementary Figure S9. The RADICL-seq data for various proteins and experimental phases, namely 2FA, NPM, and 2FA-no-NPM (representing triads present in 2FA but not in NPM). **(A-C)** The ratio of shared contacts in triads for the RADICL-seq 2FA experiment **(A)**, RADICL-seq NPM experiment **(B)**, and RADICL-seq 2FA without RADICL-seq NPM **(C)** is shown. The heatmaps are asymmetrical because of the normalization process by RDt-contacts set magnitude. This means that the magnitudes of horizontal triads datasets is divided by the magnitudes of the corresponding datasets on the vertical axis. Values greater than 1 are coloured the same as those equal to 1. If a value here is greater than 1, this means that the horizontal triads dataset is a subset of a vertical one. **(D-F)** Correlation coefficients of DNA-parts tracks of the contacts for the RADICL-seq 2FA experiment **(D)**, RADICL NPM experiment **(E)**, and RADICL-seq 2FA without RADICL-seq NPM **(F)** are shown. **(G, H)** RDt-scaling plots for cis-contacts of the RADICL-seq experiment **(G)** and for RADICL-seq-based triads **(H)**. **(I)** Ratios of ChromHMM states distribution of DNA-parts for RADICL-seq 2FA, RADICL-seq NPM and RADICL-seq 2FA without RADICL-seq NPM experiments.

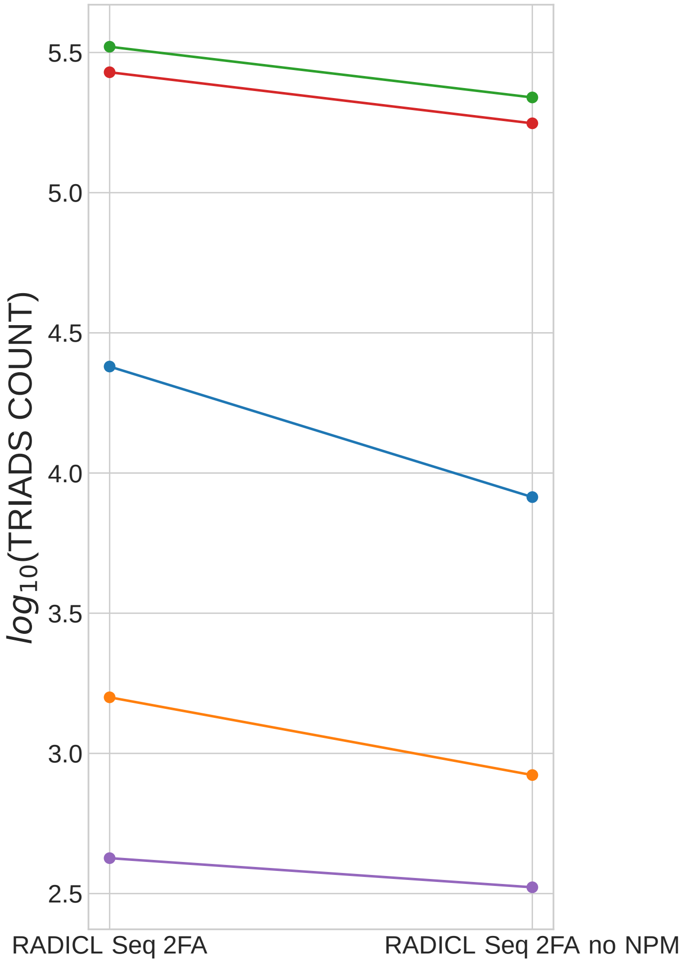

Supplementary Figure S10. Triads number desaturation when filtering the 2FA contacts shared with NPM phase.

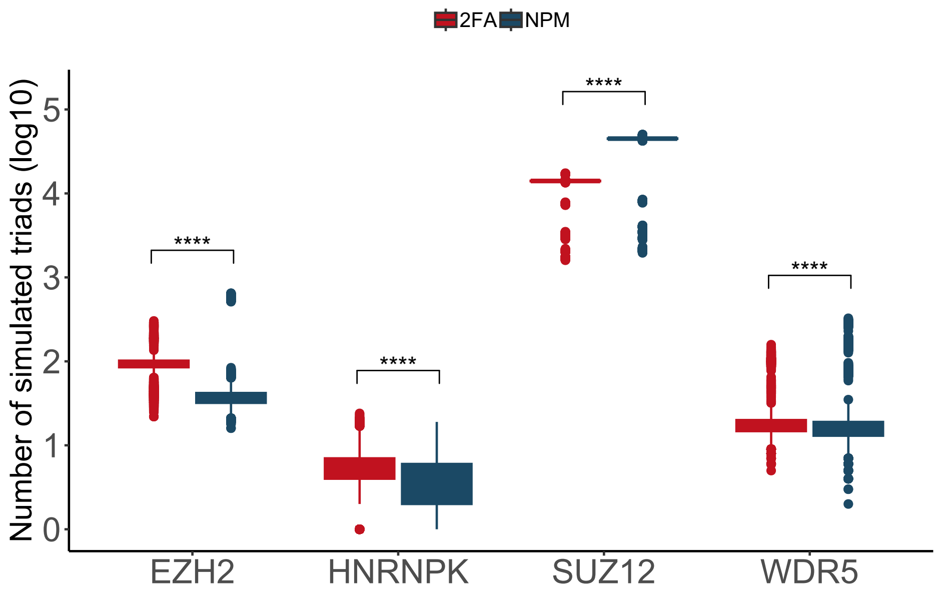

Supplementary Figure S11. Number of triads simulated for RADICL-seq 2FA and RADICL-seq NPM datasets differs significantly with the latter number being larger.

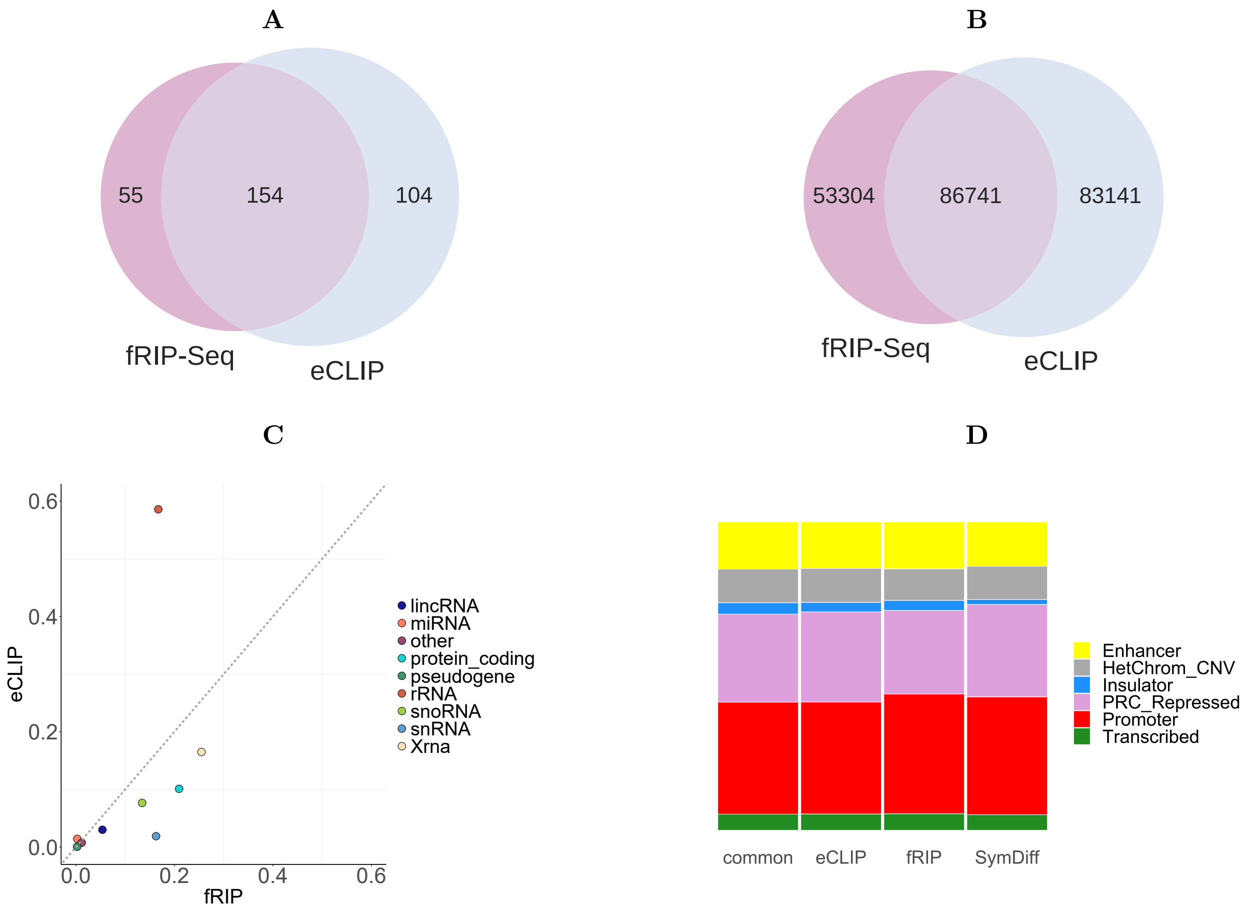

Supplementary Figure S12. Comparison of SUZ12-mediated triads based on eCLIP- and fRIP-seq PR-data. **(A, B)** Euler's diagrams for common RNAs **(A)** and shared RD-contacts **(B)** for these datasets (a contact from one dataset is considered shared if its DNA-part intersects with any DNA-part of at least one contact in the opposing dataset and RNA-parts of these contacts belong to the same transcript). **(C)** RNA biotype composition comparison of the transcripts from which the RNA parts of triads originated. **(D)** Ratios of ChromHMM states distribution for DNA-parts of SUZ12-mediated triads based on eCLIP and fRIP-seq data.

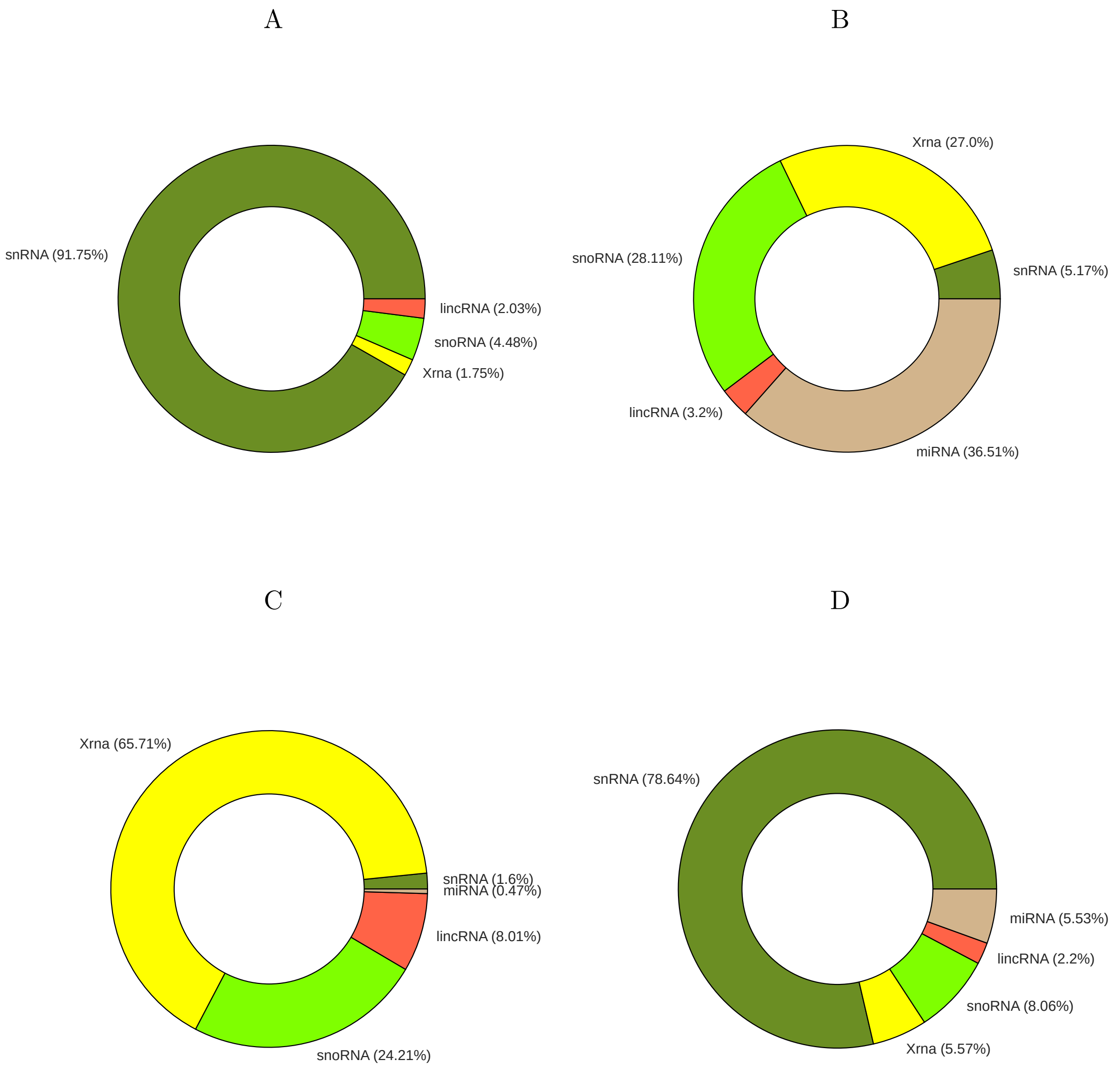

Supplementary Figure S13. Biotype content of RNAs forming triads in all fRIP-based triads **(A)**, all eCLIP-based triads **(B)**, triads shared by two experiments **(C)** and triads present only in one of datasets **(D)**.

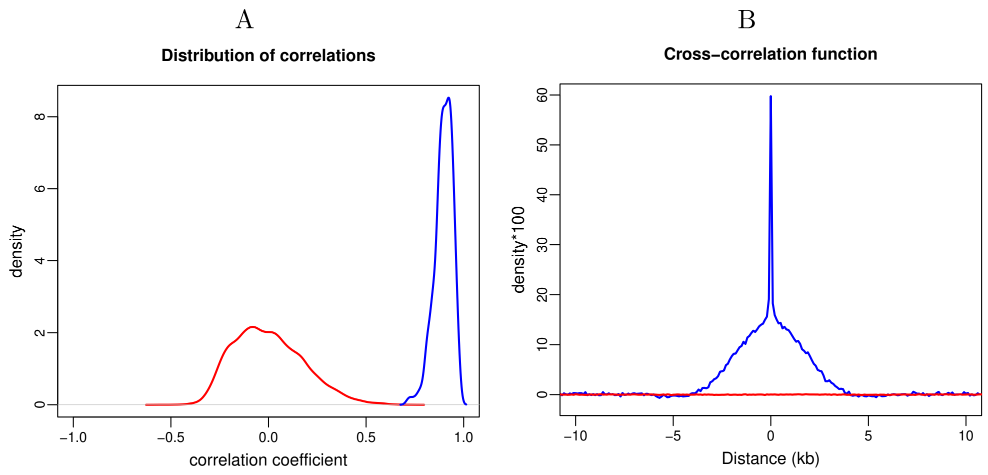

Supplementary Figure S14. SUZ12 DNA-parts tracks correlation. **(A)** Distribution of correlations across all bins; **(B)** Cross-correlation function decline. blue — observed correlation; red — background correlations

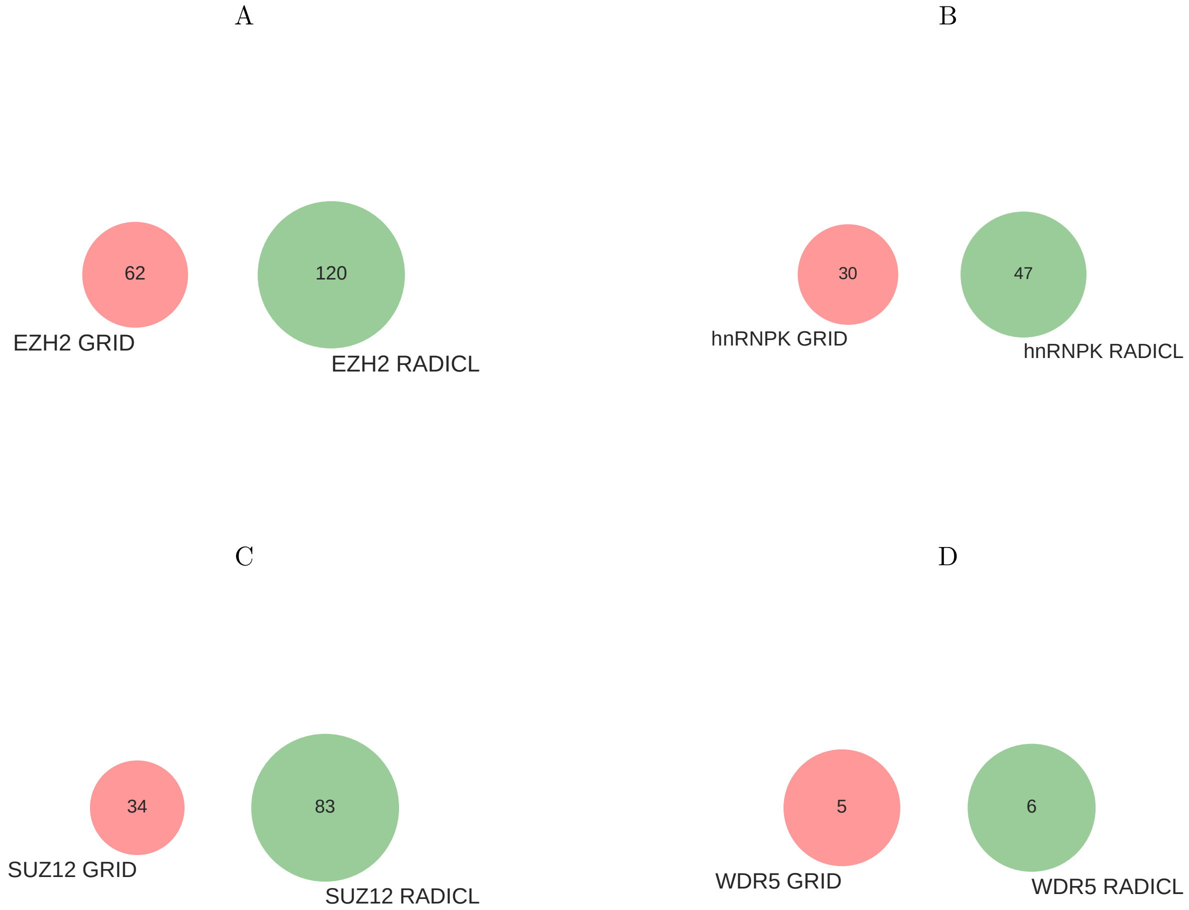

Supplementary Figure S15. Common RNA between contacts of RADICL-seq and GRID-seq in triads formed by EZH2 **(A)**, hnRNPK **(B)**, SUZ12 **(C)** and WDR5 **(D)** proteins.

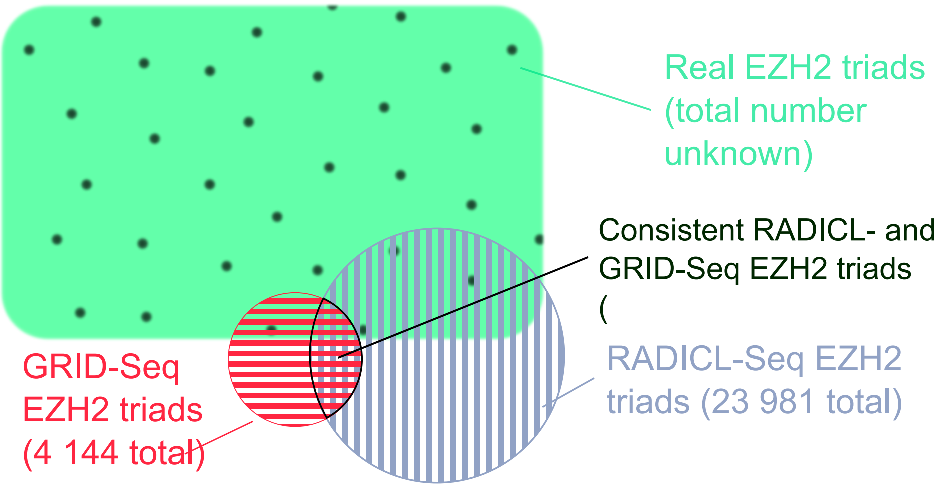

Supplementary Figure S16. Consistency analysis for EZH2-mediated triads constructed on RADICL-seq and GRID-seq data. While we can't ensure the completeness of both datasets due to experimental protocols specifics, triads shared between two experiments might be interesting to study. It is important to note, however, that the consistent triads and real EZH2-mediated triads sets might also have no objects in common.

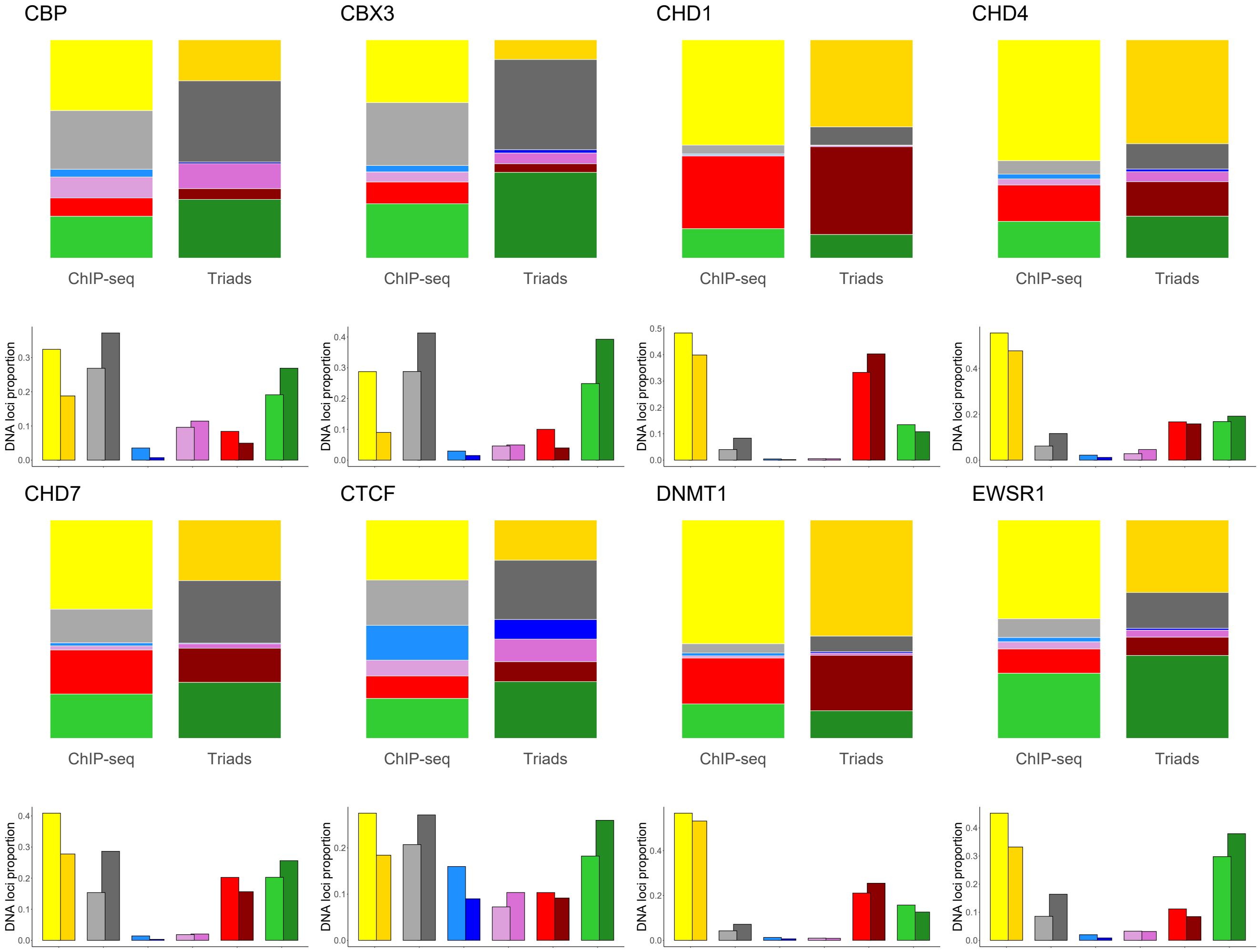

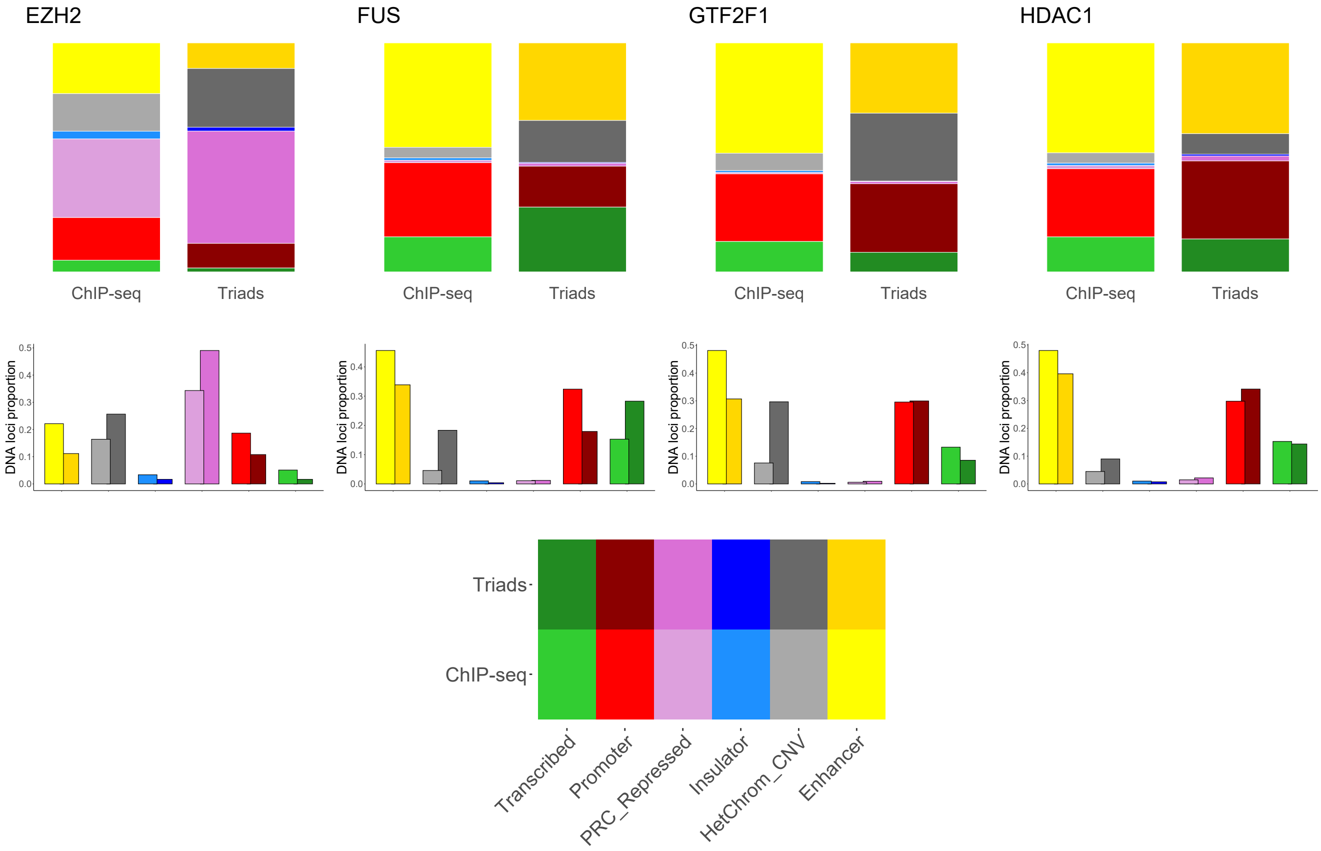
EZH2
FUS
GTF2F1
HDAC1
ChIP-seq
Triads
DNA loci proportion
Triads
ChIP-seq
Transcribed
Promoter
PRC_Repressed
Insulator
HetChrom_CNV
Enhancer

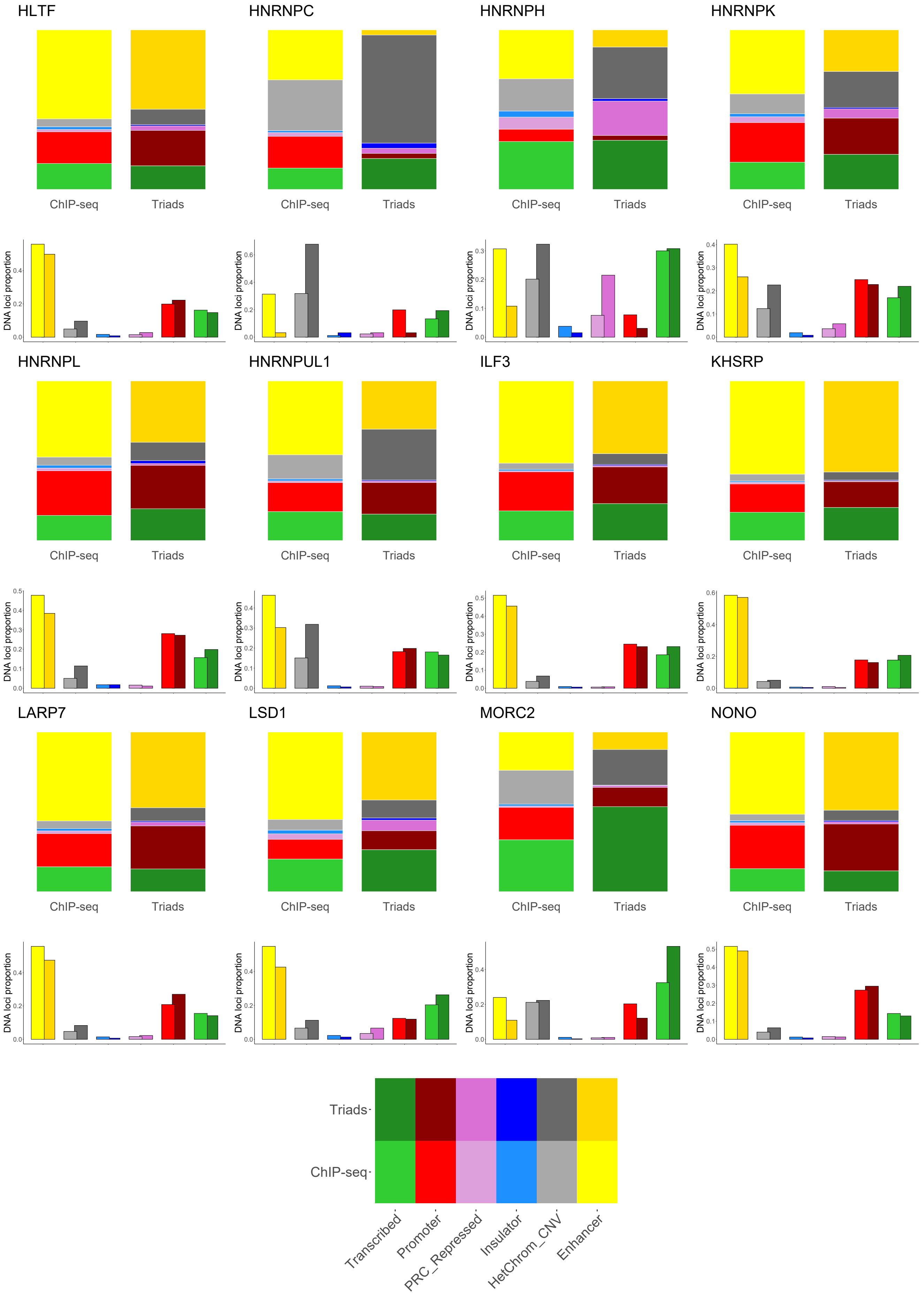
HLTF
HNRNPC
HNRNPH
HNRNPK
HNRNPL
HNRNPUL1
ILF3
KHSRP
LARP7
LSD1
MORC2
NONO
ChIP-seq
Triads
DNA loci proportion
Transcribed
Promoter
PRC_Repressed
Insulator
HetChrom_CNV
Enhancer

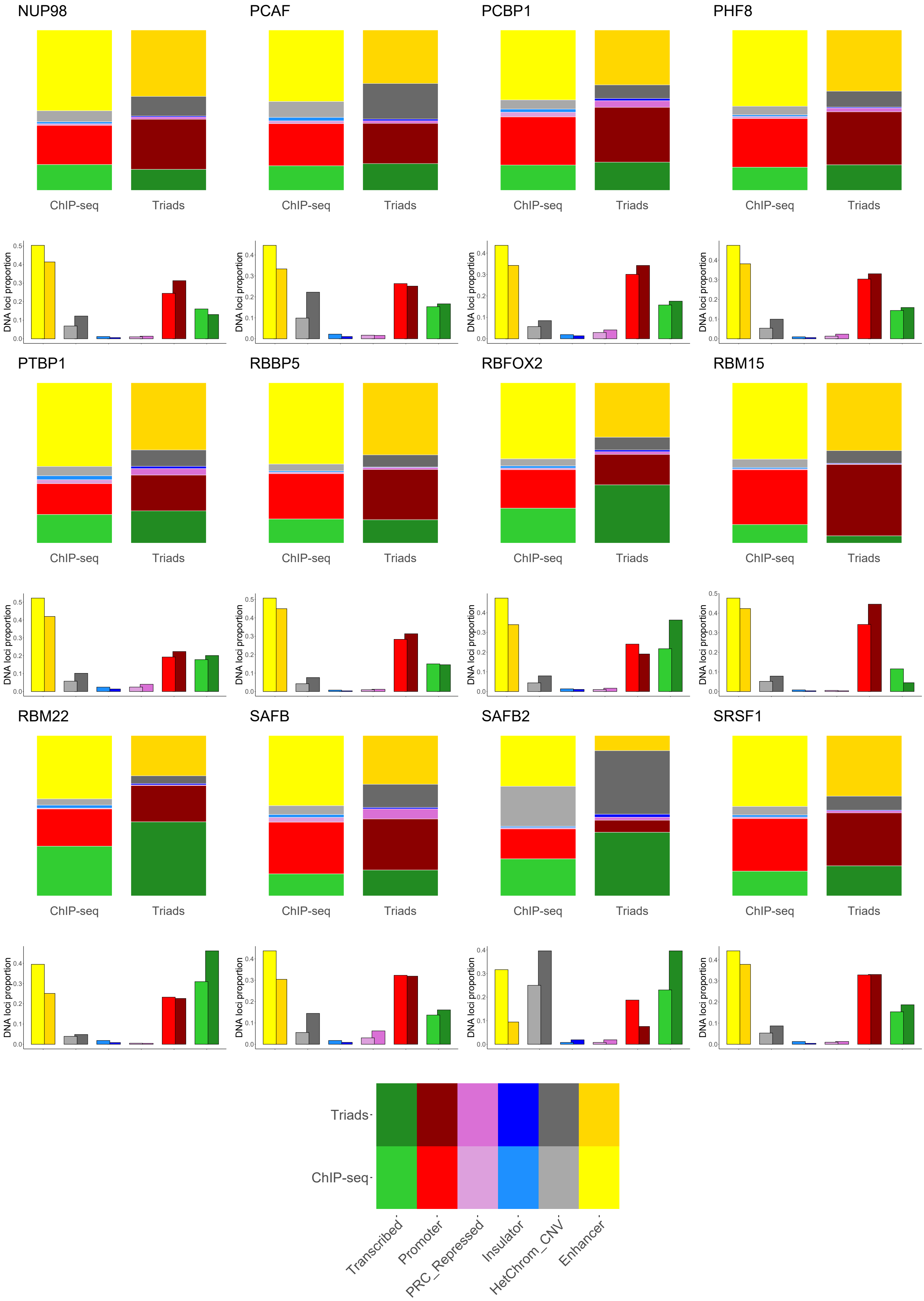
NUP98
PCAF
PCBP1
PHF8
PTBP1
RBBP5
RBFOX2
RBM15
RBM22
SAFB
SAFB2
SRSF1
ChIP-seq
Triads
DNA loci proportion
Triads
ChIP-seq
Transcribed
Promoter
PRC_Repressed
Insulator
HetChrom_CNV
Enhancer

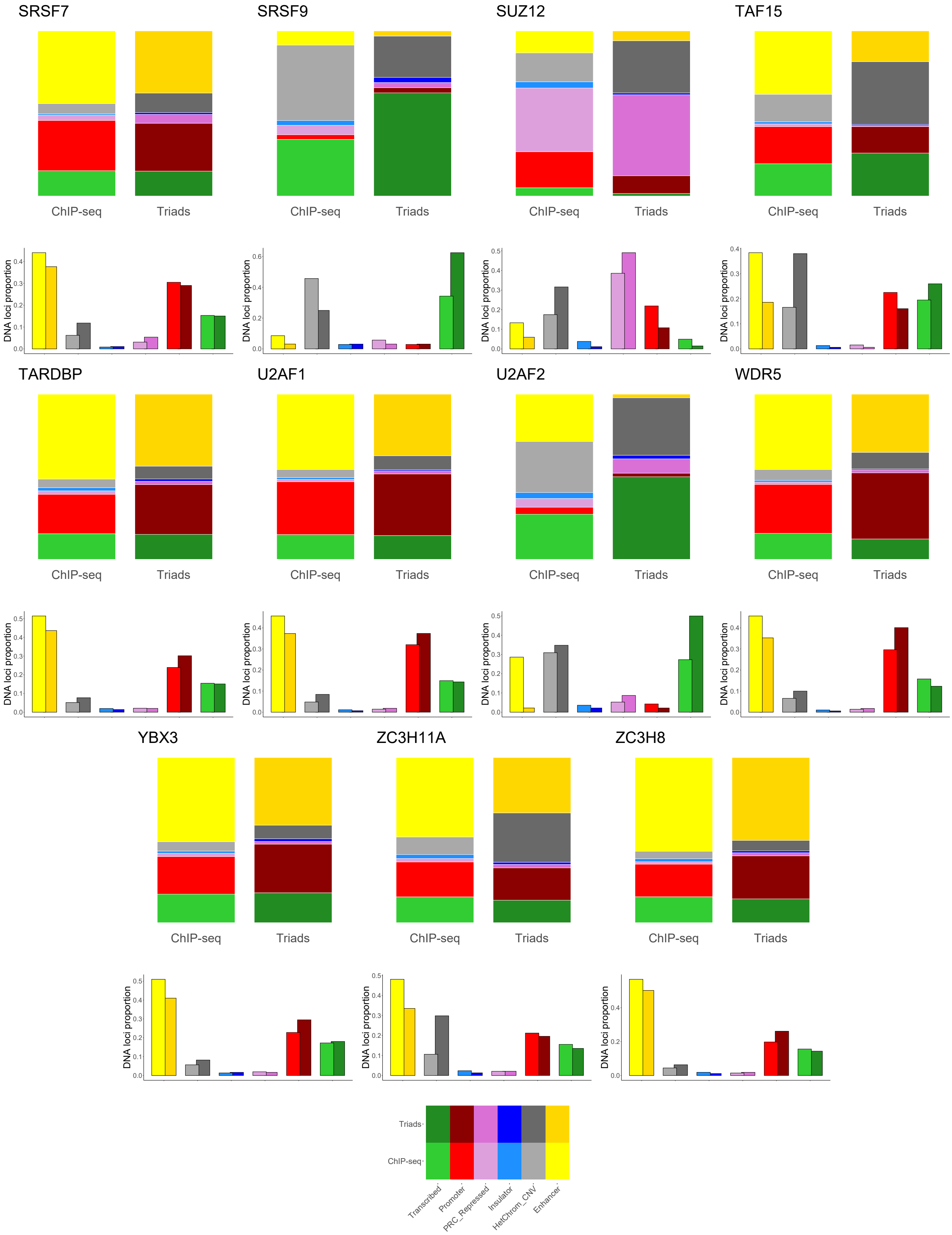

Supplementary Figure S17. Stacked barplots and paired barplots representing DNA parts distribution along the ChromHMM annotation states for triads of proteins in both cell lines.

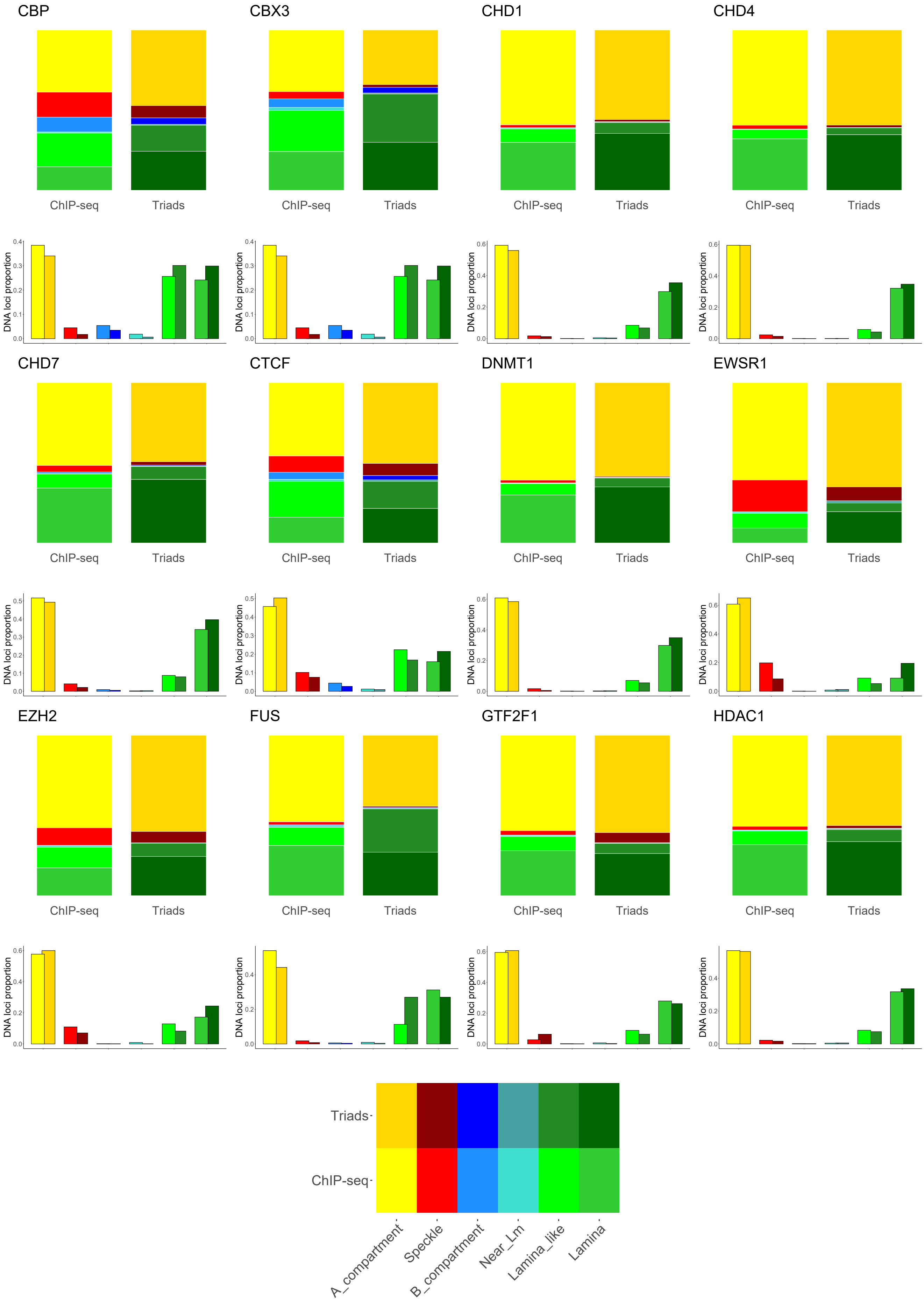
CBP
CBX3
CHD1
CHD4
CHD7
CTCF
DNMT1
EWSR1
EZH2
FUS
GTF2F1
HDAC1
ChIP-seq
Triads
DNA loci proportion
Triads
ChIP-seq
A_compartment
Speckle
B_compartment
Near_Lm
Lamina_like
Lamina

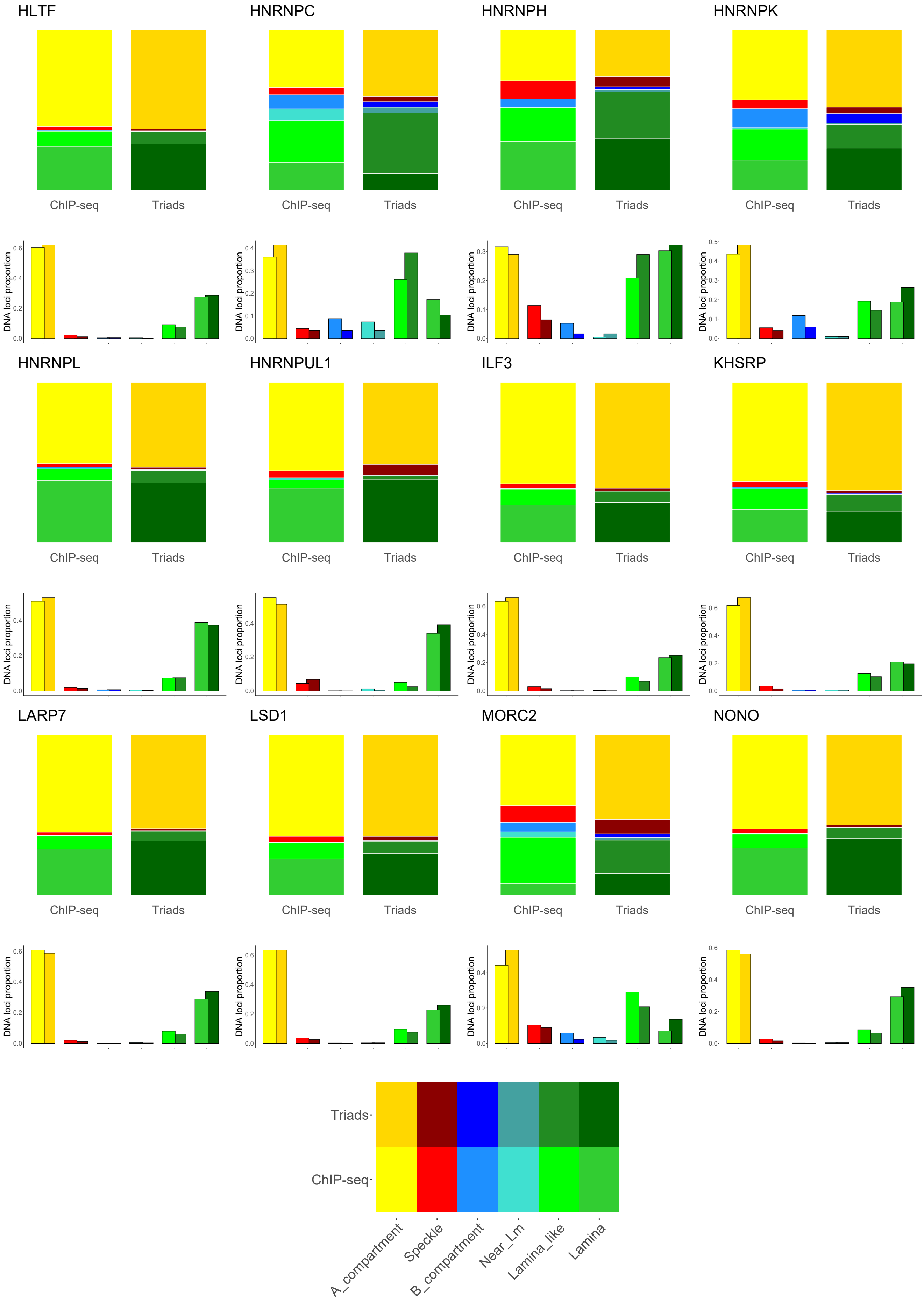
HLTF
HNRNPC
HNRNPH
HNRNPK
HNRNPL
HNRNPUL1
ILF3
KHSRP
LARP7
LSD1
MORC2
NONO
ChIP-seq
Triads
DNA loci proportion
A_compartment
Speckle
B_compartment
Near_Lm
Lamina_like
Lamina

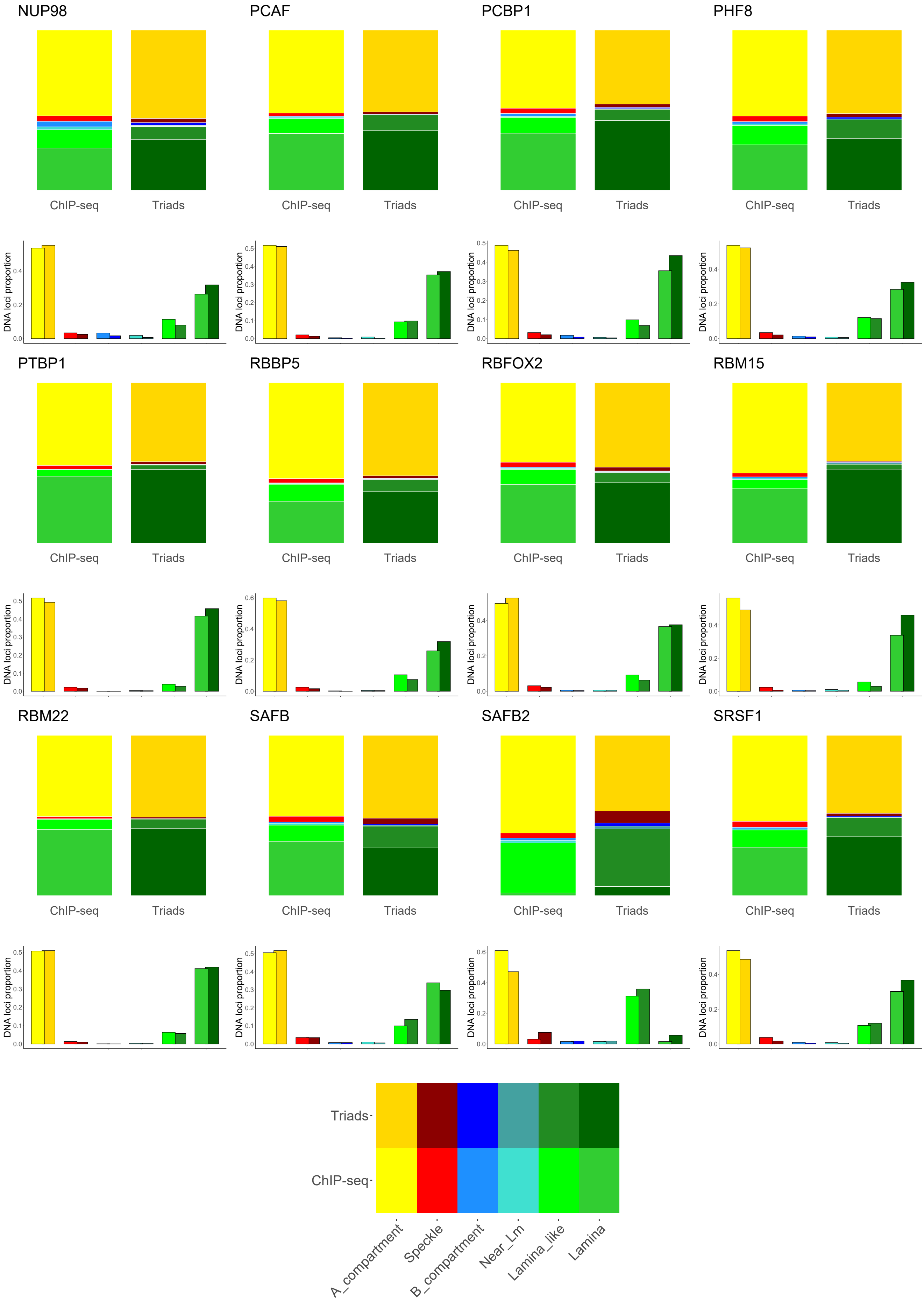
NUP98
PCAF
PCBP1
PHF8
PTBP1
RBBP5
RBFOX2
RBM15
RBM22
SAFB
SAFB2
SRSF1
ChIP-seq
Triads
DNA loci proportion
A_compartment
Speckle
B_compartment
Near_Lm
Lamina_like
Lamina

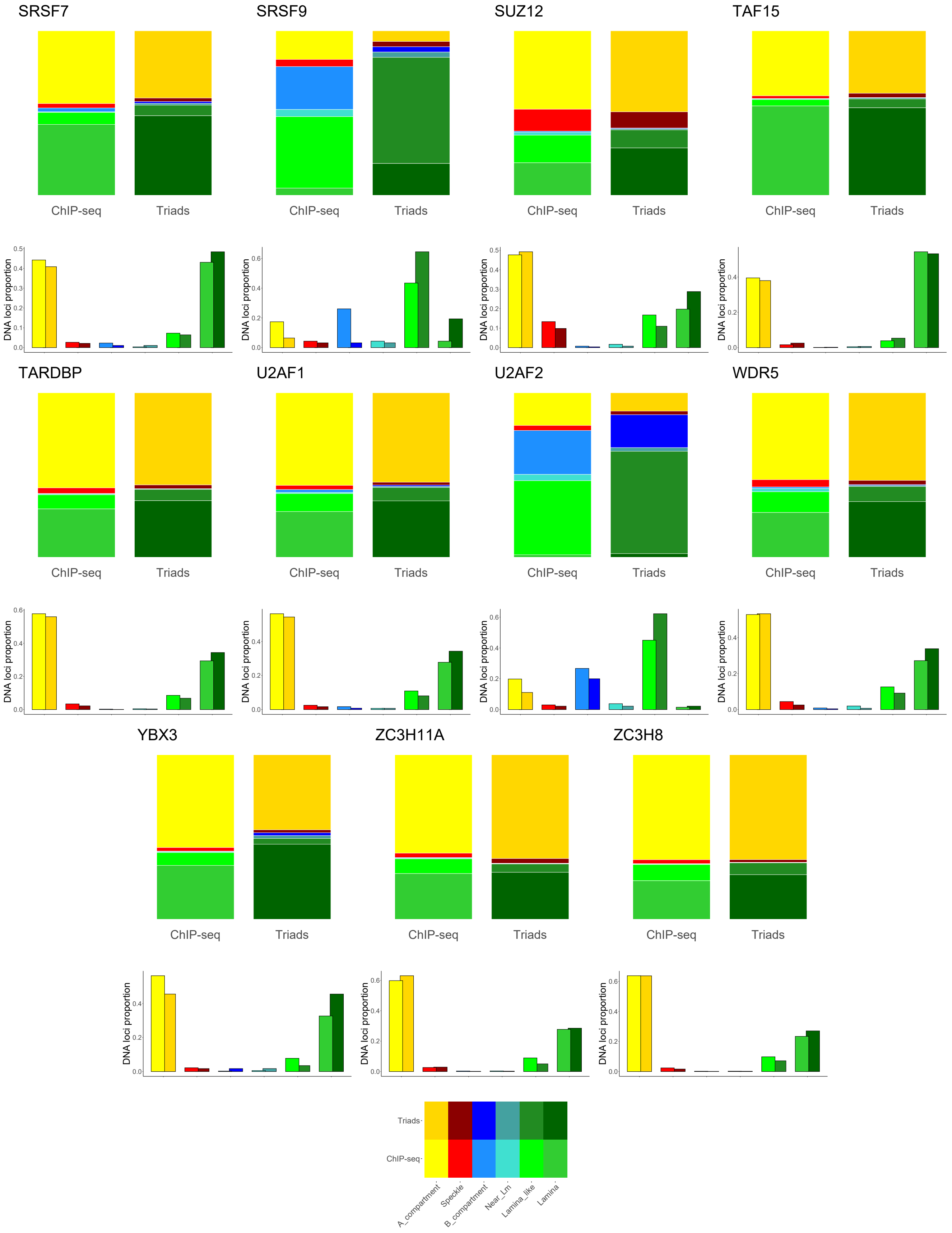

Supplementary Figure S18. Stacked barplots and paired barplots representing DNA parts distribution along the SPIN annotation states for triads of proteins in both cell lines.

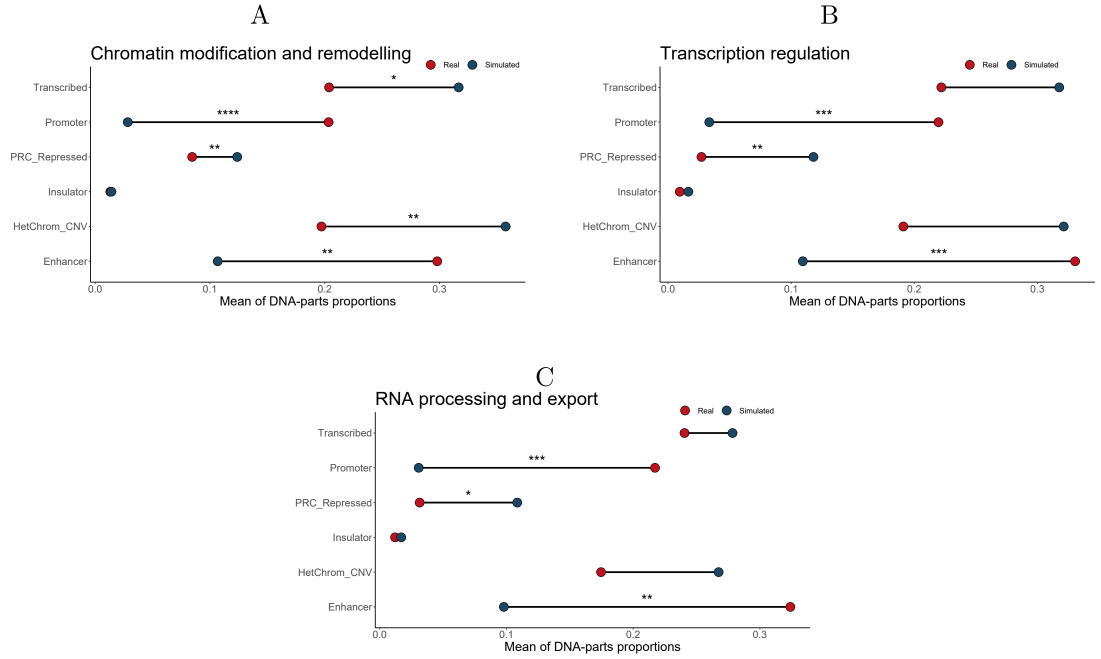

Supplementary Figure S19. DNA parts of triads distribution by ChromHMM states obtained using real and simulated DNA- and RNA-protein interaction data for RNA processing and exporting proteins.

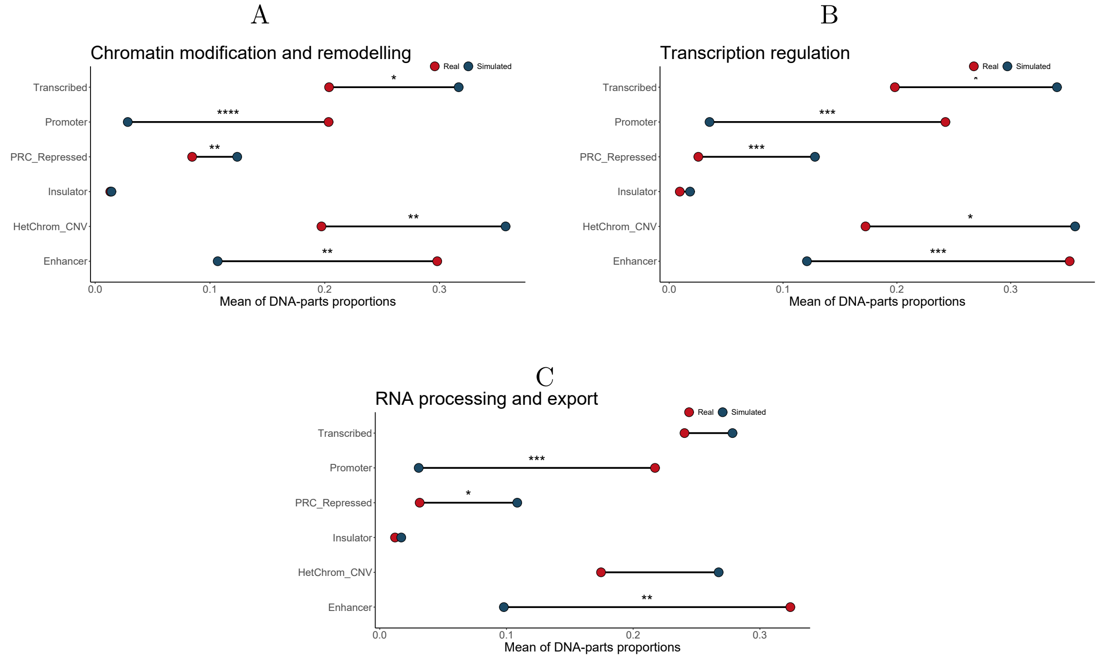

Supplementary Figure S20. DNA parts of triads distribution by ChromHMM states obtained using real and simulated DNA- and RNA-protein interaction data for RNA processing and exporting proteins. Proteins are grouped so that there are no intersections between groups.

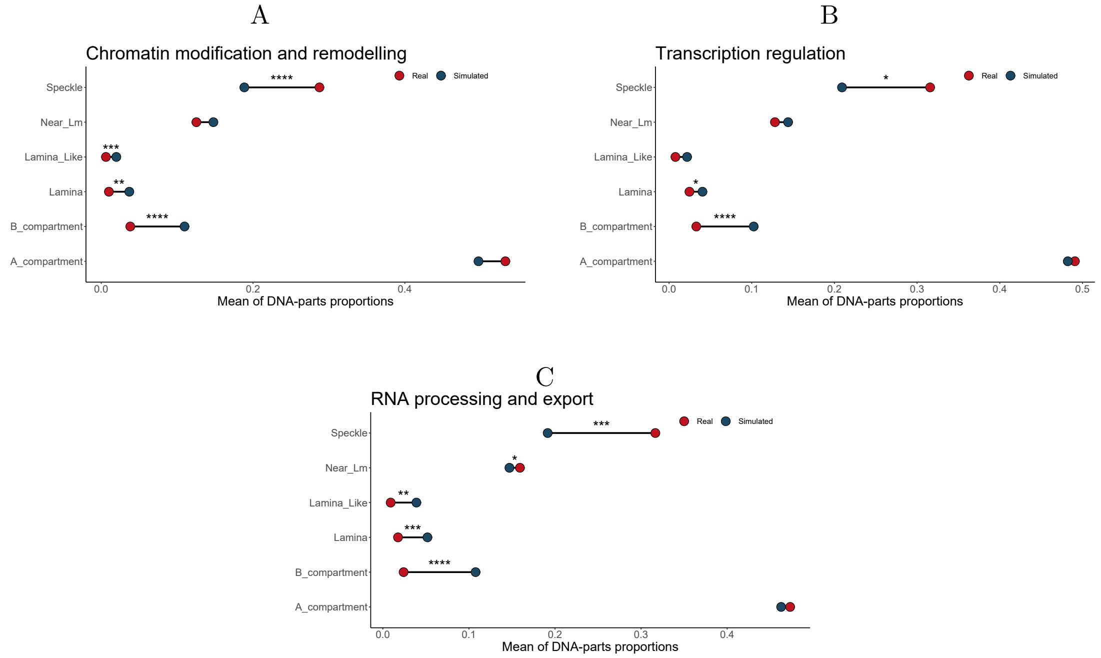

Supplementary Figure S21. DNA parts of triads distribution by SPIN states obtained using real and simulated DNA- and RNA-protein interaction data.

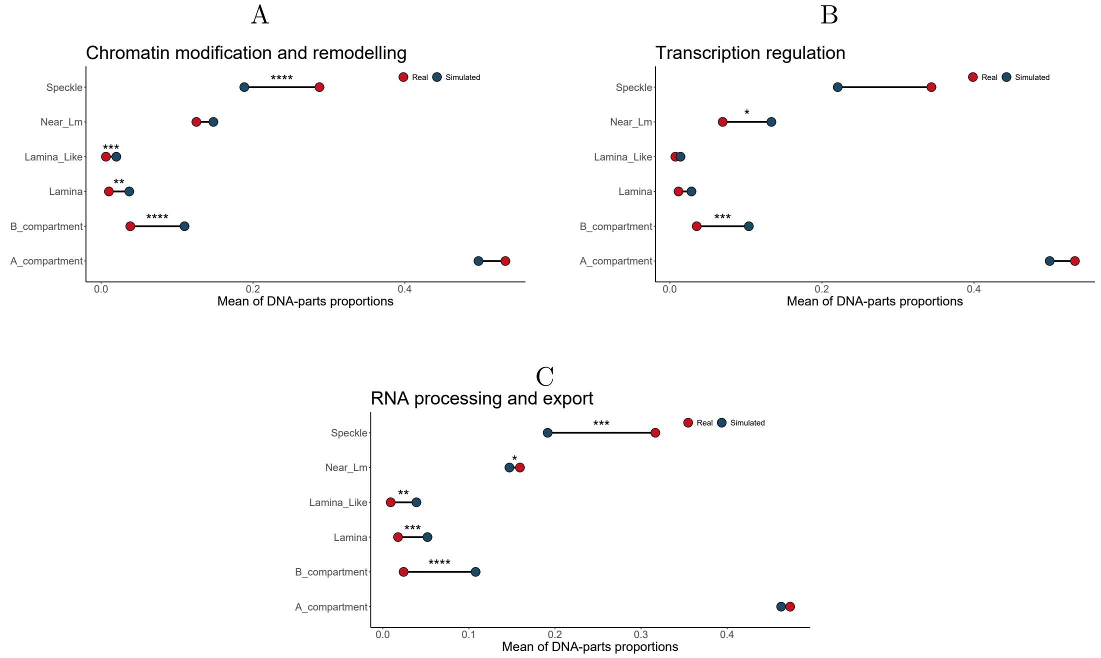

Supplementary Figure S22. DNA parts of triads distribution by SPIN states obtained using real and simulated DNA- and RNA-protein interaction data. Proteins are grouped so that there are no intersections between groups.

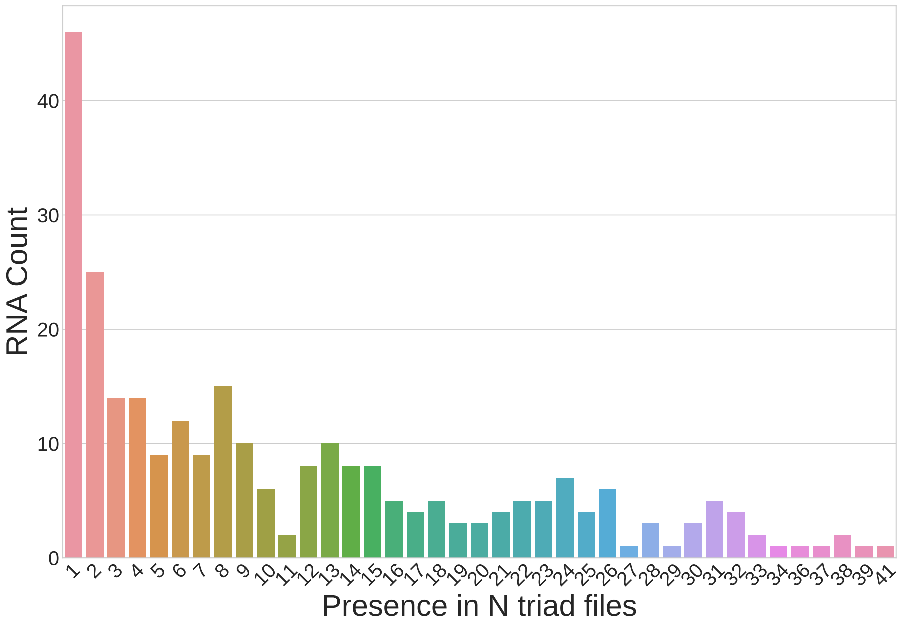

Supplementary Figure S23. RNA participation in triads, i.e. the possible range of proteins number an RNA forms triads with.
